# Structural Relevance of Intramolecular H-Bonding in *Ortho*-Hydroxyaryl Schiff Bases: The Case of 3-(5-bromo-2-hydroxybenzylideneamino) Phenol

**DOI:** 10.3390/molecules26092814

**Published:** 2021-05-10

**Authors:** İsa Sıdır, Yadigar Gülseven Sıdır, Sándor Góbi, Halil Berber, Rui Fausto

**Affiliations:** 1 Department of Physics, Faculty of Science and Arts, Bitlis Eren University, 13000 Bitlis, Turkey; yadigar.gulseven@gmail.com; 2 CQC, Department of Chemistry, University of Coimbra, 3004-535 Coimbra, Portugal; sandor.gobi@gmail.com; 3 MTA-ELTE Lendül the Four Most Stable Conformers of the et Laboratory Astrochemistry Research Group, Institute of Chemistry, ELTE Eötvös Loránd University, H-1518 Budapest, Hungary; 4 Department of Chemistry, Faculty of Science, Eskişehir Technical University, 26470 Eskişehir, Turkey; hlberber@eskisehir.edu.tr

**Keywords:** Schiff base, conformational space, matrix isolation infrared spectroscopy, intramolecular hydrogen bonding, DFT/B3LYP/6-311++G(d,p) calculations, quantum mechanical tunneling, NBO analysis

## Abstract

A new Schiff base compound, 3-(5-bromo-2-hydroxybenzylideneamino)phenol (abbreviated as BHAP) was synthesized and characterized by ^1^H- and ^13^C- nuclear magnetic resonance and infrared spectroscopies. DFT/B3LYP/6-311++G(d,p) calculations were undertaken in order to explore the conformational space of both the *E*- and *Z*- geometrical isomers of the enol-imine and keto-amine tautomers of the compound. Optimized geometries and relative energies were obtained, and it was shown that the most stable species is the *E*-enol-imine form, which may exist in four low-energy intramolecularly hydrogen-bonded forms (I, II, V, and VI) that are almost isoenergetic. These conformers were concluded to exist in the gas phase equilibrium with nearly equal populations. On the other hand, the infrared spectra of the compound isolated in a cryogenic argon matrix (10 K) are compatible with the presence in the matrix of only two of these conformers (conformers II and V), while conformers I and VI convert to these ones by quantum mechanical tunneling through the barrier associated with the rotation of the OH phenolic group around the C–O bond. The matrix isolation infrared spectrum was then assigned and interpreted with help of the DFT(B3LYP)/6-311++G(d,p) calculated infrared spectra for conformers II and V. In addition, natural bond orbital (NBO) analysis was performed on the most stable conformer of the experimentally relevant isomeric form (*E*-enol-imino conformer V) to shed light on details of its electronic structure. This investigation stresses the fundamental structural relevance of the O–H···N intramolecular H-bond in *o*-hydroxyaryl Schiff base compounds.

## 1. Introduction

**Scheme 1:**
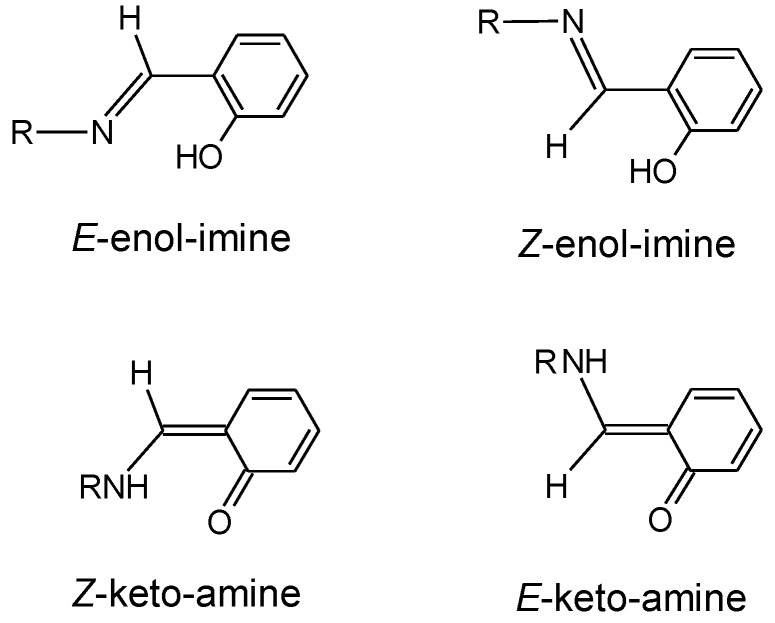
Relevant tautomeric forms of *o*-hydroxyaryl Schiff bases. The keto-amine tautomer exists in the *Z* and *E* isomers as shown in the left and right bottom images, respectively. However, the geometric isomers of this tautomer do not have a direct correspondence to the geometric isomers of the enol-imine tautomer. To avoid confusion, in this article the keto-amine conformers will be grouped in two sets, one related to the *E*-enol-imine tautomer and other to the *Z*-enol-imine tautomer, which will be designated as “keto-amine forms of the *E*-enol-imine tautomer” and “keto-amine forms of the *Z*-enol-imine tautomer”, respectively. In both sets, there are *Z*-keto-amine and *E*-keto-amine conformers.

*Ortho*-hydroxyaryl Schiff bases are compounds structurally described by having an *ortho* hydroxyl-substituted aromatic ring connected to the carbon atom of the Schiff base characteristic azomethine group (–CH=N−) (Scheme 1). These compounds have a wide range of practical applications, such as in optical data storage devices [1,2,3,4,5], molecular switches [6,7], and sensors [8,9], and are also highly valued for micro- and nanostructures’ fabrication, since they are versatile templates for molecular assembling [10,11,12,13].

The applications of this type of Schiff base are mostly determined by specific interactions involving their –CH=N− and phenolic *o-*hydroxyl substituent moieties, in particular the characteristic O–H···N intramolecular H-bond interaction between these two groups that facilitates intramolecular proton transfer and tautomerization [14,15,16,17,18]. Two relevant tautomeric forms of the compounds thus exist (enol-imine and keto-amine forms; see Scheme 1), which in general are easily interconvertible either thermally or photochemically. In turn, both enol-imine and keto-amine tautomers have two geometric isomers (*E* and *Z* forms), defined around the exocyclic C=N (in the enol-imine tautomer) or C=C (in the keto-amine tautomer) bond, which may also possess several conformational isomers.

The intramolecular H-bonding in *o*-hydroxyaryl Schiff bases has been shown to be very much sensitive to the characteristics of the substituents present in the phenolic and azomethine groups, with bulky substituents forcing these groups to lost co-planarity and leading to a remarkable lengthening and weakening of the H-bond [19,20,21]. The intramolecular proton transfer in this type of compounds depends also on solvent polarity and temperature [22,23], while optical excitation of the intramolecularly H-bonded Schiff base can lead to excited state intramolecular proton transfer (ESIPT) [24,25,26,27], which may also be accompanied by isomerization around the azomethine bond [28,29,30].

One of the most interesting properties that is in general exhibited by *o*-hydroxyaryl Schiff base derivatives is solvatochromism, which ultimately is a manifestation of the relative populations of the enol-imine and keto-amine tautomers present in the different media [14,15,16,17]. Most of times, solvatochromism is investigated by room temperature electronic absorption and fluorescence emission spectroscopies, and the simultaneous presence of several isomers and conformers of each tautomer in the samples has been considered a factor leading to an increased complexity in the analysis of the experimental data [15,16,17]. Hence, creating conditions where the number of conformers might be reduced appears of interest to undertake a detailed structural characterization of the tautomeric forms. On the other hand, the understanding of the main characteristics of the conformational space of this type of compounds is instrumental for the understanding of their properties. For this purpose, minimization of the intermolecular interactions is of fundamental importance.

Matrix isolation is an experimental sampling technique that suits all the requirements stated above. In one side, being a low temperature technique (the work temperature is usually of a few degrees Kelvin), conditions are created that allow reducing the number of conformers present in the samples, and, on the other side, intermolecular interactions between the matrix-isolated molecules and the inert matrix medium (in general a frozen noble gas, e.g., argon) are minimal [31,32,33,34]. Matrix isolation has also many practical advantages compared with other possible approaches (e.g., molecular beams), in particular when the probing method is a spectroscopic technique [31,32]. Moreover, the concerted use of matrix isolation infrared spectroscopy and quantum chemical electronic structure calculations has been proven to be a very powerful tool for structural studies on conformationally flexible and multi-isomeric complex systems [31,32,33,34,35,36,37].

**Figure 1:**
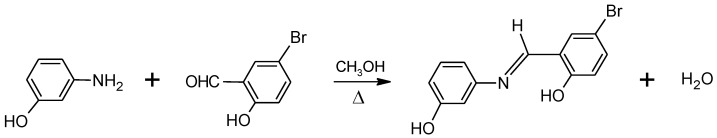
Synthesis scheme of 3-(5-bromo-2-hydroxybenzylideneamino)phenol (BHAP). The product is drawn in the *E*-enol-imine form (see Section 3, for discussion).

The study reported herein focused on 3-(5-bromo-2-hydroxybenzylideneamino)phenol (abbreviated as BHAP, Figure 1), which is a conformationally flexible *o*-hydroxyaryl Schiff base that exhibits tautomerism and geometric isomerism (*E*/*Z*). The conformational spaces of the two main tautomers of the molecule were investigated using electronic structure quantum chemical calculations undertaken at the DFT/B3LYP/6-311++G(d,p) level of theory, and the compound was experimentally studied under matrix isolation conditions by infrared spectroscopy. As it will be described in details in the next sections, it was concluded that the most stable species of BHAP is its *E*-enol-imine form, which has 4 low-energy intramolecularly hydrogen-bonded forms (I, II, V and VI) which are of experimental relevance. These four conformers are concluded to exist in the gas phase with nearly equal populations, while only two of them (II and V) could be efficiently trapped in a cryogenic (10 K) argon matrix. Conformers I and VI convert to conformer II and V, respectively, by quantum mechanical tunneling through the barrier associated with the rotation of the OH phenolic group around the C–O bond. Natural bond orbital (NBO) analysis was performed on the most stable conformer of the experimentally relevant isomeric form (*E*-enol-imino conformer V) and used to shed light on details of its electronic structure. As a whole, this investigation is a comprehensive structural study of BHAP, and stresses the relevance of the O–H···N intramolecular interaction in determining the structure and properties of *o*-hydroxyaryl Schiff base compounds.

## 2. Experimental Procedures and Computational Methods

### 2.1. Synthesis

3-(5-Bromo-2-hydroxybenzylideneamino)phenol was synthesized by mixing the equivalent amounts of the substituted amine (3-aminophenol) and substituted aldehyde (5-bromo-2-hydroxybenzaldehyde) precursors in methanol, at ca. 45 °C (see Figure 1) [15,16,17]. 3-Aminophenol (1.091 g; 0.01 mol) and 5-bromo-2-hydroxybenzaldehyde (2.000 g; 0.01 mol) were first dissolved in 25 mL of methanol by heating, and the solutions were then added. After 1 h at ca. 45 °C, under slow stirring, the reaction completed and the precipitated product was filtered, purified by recrystallization from methanol, and dried in a vacuum desiccator at room temperature. The IR spectrum of the purified crystalline material (in a KBr pellet at room temperature), and the ^1^H- and ^13^C-NMR spectra in dimethylsulfoxide (DMSO-d_6_) solution were found to be compatible with the desired product, and are provided in Supplementary Figures S1–S3. The IR spectrum was obtained in a Perkin Elmer FT-IR 100 spectrometer, and the ^1^H- and ^13^C-NMR spectra were obtained using a Bruker Biospin Ultrashield^TM^ 300 MHz NMR spectrometer (Bruker, Karlsruhe, Germany), at room temperature. The melting point (m. p.) of the compound was measured using a Gallenkamp Sanyo Heater.

IR (KBr disc, ν in cm^−1^): ~3322–2600 (O–H), ~3085–3000 (C–H, aromatic), 1625 (N=C), 1587–1469 (C=C, aromatic), 1326/1244 (C–O). ^1^H NMR (300 MHz, DMSO-d_6_) δ 13.15 (s, 1H), 9.70 (s, 1H), 8.88 (s, 1H), 7.86 (d, *J* = 2.5 Hz, 1H), 7.53 (dd, *J* = 8.8, 2.6 Hz, 1H), 7.25 (t, *J* = 7.9 Hz, 1H), 6.94 (d, *J* = 8.8 Hz, 1H), 6.87–6.72 (m, 3H). ^13^C NMR (75 MHz, DMSO-d_6_) δ 162.11 (s), 159.88 (s), 158.80 (s), 149.45 (s), 135.89 (s), 134.50 (s), 130.69 (s), 121.57 (s), 119.48 (s), 114.88 (s), 112.63 (s), 110.33 (s), 108.60 (s). Elemental analysis, (calculated for C_13_H_10_BrNO_2_)/observed using a CNHS-932 LECO apparatus: C, (53.450)/51.380; H, (3.450)/3.846; N, (4.795)/4.635. Solid, m. p. 160–163 °C.

### 2.2. Matrix-Isolation Experiments

To prepare the cryogenic matrices, BHAP was placed in a homemade Knudsen cell assembled inside the vacuum chamber of the cryostat (APD Cryogenics closed-cycle helium refrigeration system with a DE202 expander), and was sublimated through thermoelectrical heating with help of a DC Power Supply VITEECOM (model 75-HY5003). The vapor of the compound was then co-deposited with argon (Air Liquide, N60), in a ~1:1000 solute:host molar ratio, onto the optical CsI substrate of the cryostat, kept at 10.0 ± 0.1 K. 

The IR spectra of the matrices were obtained using a Nicolet 6700 FTIR spectrometer, equipped with a mercury cadmium telluride (MCT) detector and a KBr beam splitter. The IR spectra were recorded in the 4000–500 cm^−1^ range, with 0.5 cm^−1^ resolution. The instrument was purged by a stream of dry/CO_2_-filtered air in order to avoid interference from atmospheric H_2_O and CO_2_ vapors. 

### 2.3. Theoretical Calculations

**Figure 2:**
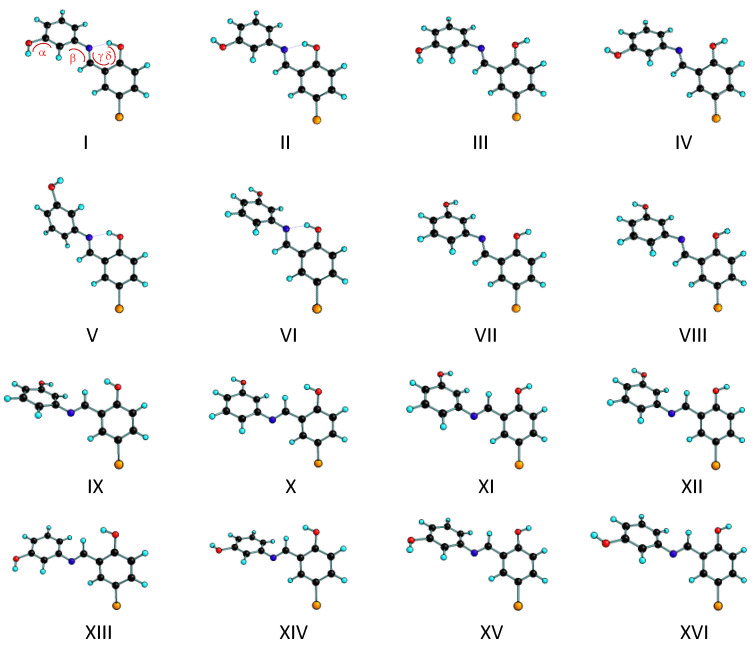
Conformers of the BHAP *E*-enol-imine form. The B3LYP/6-311++G(d,p) calculated optimized Cartesian coordinates of each conformer are provided in the Supporting Information Table S1. The conformationally relevant dihedral angles α, β, ϒ, δ are indicated over the structure of conformer I. Note that all conformers have a symmetry related structure.

**Figure 3:**
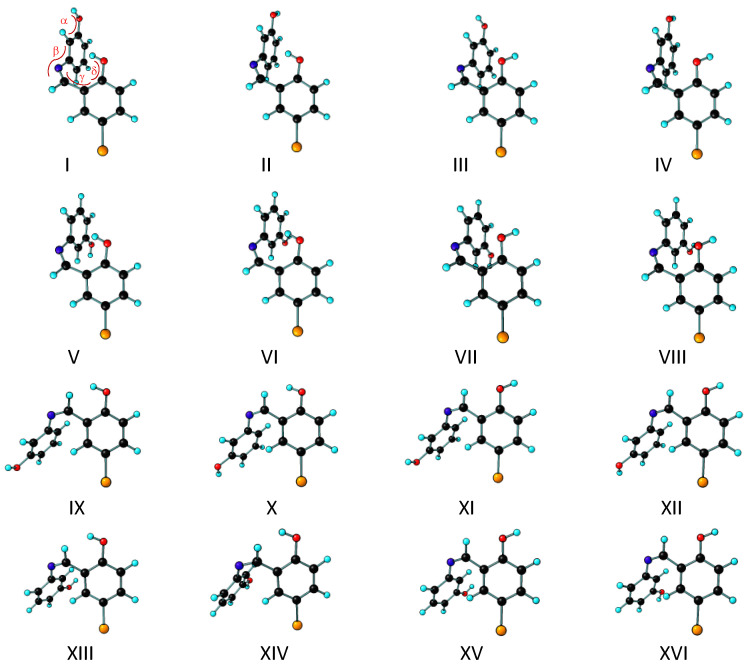
Conformers of the BHAP *Z*-enol-imine form. The B3LYP/6-311++G(d,p) calculated optimized Cartesian coordinates of each conformer are provided in the Supporting Information Table S1. The conformationally relevant dihedral angles α, β, ϒ, δ are indicated over the structure of conformer I. Note that all conformers have a symmetry related structure.

**Figure 4:**
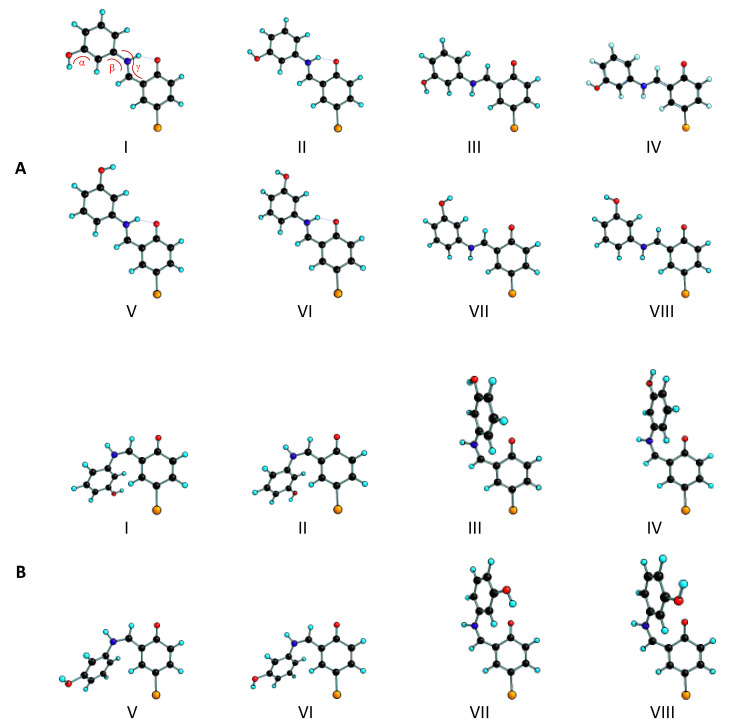
Conformers of the BHAP keto-amine forms: (**A**), keto-amine forms structurally related to the *E*-enol-imine tautomer; (**B**), keto-amine forms structurally related to the Z-enol-imine tautomer. B3LYP/6-311++G(d,p) calculated optimized Cartesian coordinates of each conformer are provided in the Supporting Information Table S1. The conformationally relevant dihedral angles α, β, ϒ are indicated over the structure of conformer I (**A**). Note that all conformers have a symmetry related structure.

The geometries and IR spectra (within the harmonic approximation) of the conformers of *E*-enol-imine and *Z*-enol-imine isomers of BHAP, and the corresponding keto-amine forms (Figure 2, Figure 3 and Figure 4) were fully optimized at the DFT level of theory, with the B3LYP functional and the 6-311++G(d,p) basis set [38,39,40,41,42,43]. The harmonic vibrational frequencies were scaled by the factors 0.994 and 0.945, below and above 1800 cm^−1^, respectively, to approximately correct them for the neglected anharmonicity and method/basis set limitations. These scaling factors were used throughout the study, being applied to the calculated spectra of all structures considered. Relaxed potential energy profiles for the relevant internal rotations in the BHAP molecule were calculated at the same theory level. All calculations were performed with GAUSSIAN 09 (revision A.02) [44]. Assignment of the vibrational modes was carried out with the help of the animation module of GaussView (version 5.0) program [45]. In the simulated spectra presented in the figures, the IR bands were broadened by Lorentzian profiles (fwhm = 10 cm^−1^) centered at the calculated (scaled) frequencies, using the program ChemCraft [46].

## 3. Results and Discussion

### 3.1. Structures and Conformational Landscapes of BHAP Isomeric Forms

The two geometrical enol-imine isomers of the BHAP differ in the placement of the substituents around the azomethine C=N bond, which leads to isomers *E* and *Z*. These isomers have four conformationally relevant internal axes of rotation: two of these (α and δ in Figure 2 and Figure 3) correspond to the rotation around the two phenolic C–O bonds and define the orientation of the phenolic OH hydrogen atoms, while the remaining two axes of internal rotation (β and ϒ in Figure 2 and Figure 3) are associated with the C–N and C–C exocyclic bonds of the bridge that connects the two phenolic rings, thus determining the relative orientation of the two aromatic rings. The performed B3LYP/6-311++G(d,p) calculations predicted the existence of 16 distinct conformers for each enol-imine geometrical isomer, whose optimized structures are depicted in Figure 2 and Figure 3. The conformationally relevant dihedral angles for these conformers are given in Table 1, while the corresponding electronic energies (with and without zero-point correction), Gibbs standard energies (at 298.15 K), and dipole moments are given in Table 2.

As shown in Table 2, the B3LYP/6-311++G(d,p) calculations predicted *E*-enol-imine conformer V as the lowest energy species among all possible structures. All the conformers of the *Z*-enol-imine isomer have much higher energies than the *E*-enol-amine V form, the lowest energy *Z*-enol-imine conformer being conformer *Z*-enol-imine V, which stays 62 kJ mol^−1^ above *E*-enol-imine V. Notably, conformer *Z*-enol-imine V is in fact higher in energy than all *E*-enol-imine forms. The main reason for the high energy of the *Z*-enol-imine conformers is the repulsion between the two phenolic rings, which in the *Z*-isomer stay in the same side of the azomethine C=N bond (see Figure 3).

The four most stable conformers of the *E*-enol-imine isomer (conformers I, II, V and VI) are more than 38 kJ mol^−1^ lower in energy than the next conformer in the increasing order of energy (conformer XV). These conformers exhibit an intramolecular hydrogen-bond interaction of the O–H···N type (see Figure 2), which leads to the formation of a nearly planar pseudoaromatic six-membered ring where the dihedral angles ϒ and δ are both ~0°, and that is responsible for their stabilization [47]. Within the two pairs of conformers (I, II) and (V, VI), the main structural difference is the orientation of the non-hydrogen bonded phenolic OH group (the α dihedral angle being near 0 or 180°), while the orientation of the two aromatic rings is the same (i.e., the dihedrals β and ϒ are similar in each pair of conformers). The two pairs differ essentially in the value of the dihedral angle β, which is ca. 37° in I and II and about −144° in V and VI. Note that in all the low-energy intramolecularly H-bonded *E*-enol-imine conformers the bromo-substituted phenolic ring is roughly planar with the azomethine moiety (ϒ is ~0°) due to the involvement of these two molecular fragments in the hydrogen bond, while the non-substituted phenol ring deviates considerably from the plane formed by the bromo-substituted phenolic ring and azomethine group, mostly because of the repulsive interaction between the azomethine hydrogen atom and the closest located hydrogen atom of the unsubstituted phenolic moiety (see Figure 2).

The four hydrogen bonded *E*-enol-imine conformers have predicted energies within less than 2 kJ mol^−1^, which after zero-point and thermal corrections lead to standard Gibbs energies (at 298.15 K) that are within less than 1.5 kJ mol^−1^, i.e., in practical terms the calculations predict the four conformers as being nearly isoenergetical. The electronic energy of conformer V was predicted to be the lowest (followed by those of conformers II, VI and I, which are higher than that of conformer V by 0.10, 0.84, and 1.69 kJ mol^−1^, respectively), and inclusion of the zero-point correction does not change the order of stability of the conformers nor their relative energies significantly (see Table 2). However, conformers I and II have slightly larger entropy than forms V and VI, so that their relative standard Gibbs energies at 298.15 K comparatively reduce. In particular, this makes conformer II to become the lowest Gibbs energy form at the considered temperature. Nevertheless, all differences in the energies and entropy of the four intramolecularly H-bonded, low-energy conformers of the *E*-enol-imine isomer of the studied Schiff base are very small and, as mentioned above, in practical terms the four conformers shall be considered nearly isoenergetical and expected to be approximately equally populated in the gas phase equilibrium (i.e., each conformer shall have a population of approximately 25%, since all the other conformers have much higher energy and can be expected to have negligible populations).

The intramolecular H-bond in the four experimentally relevant *E*-enol-imine conformers (I, II, V, VI) is predicted by the calculations to be of nearly the same strength, taking into account the associated geometric parameters: in all forms, the O–H bond length is 0.993 Å (considerably longer than that of the free phenolic OH group that is 0.962 Å long), and the H···N and O···N distances are within the narrow ranges of 1.742–1.746 and 2.632–2.635 Å, respectively. The O–H···N angle is also very much similar in all the four conformers, being within the 146.9–147.1° range. In each conformer, the two C–O bonds are predicted to be considerably different from each other, with that associated with the H-bonded phenolic group being considerably shorter (1.339 Å in all the conformers) than that associated with the free OH group (1.368 Å in I and II and 1.367 Å in V and VI). The shortening of both C–O and O–H bonds in the H-bonded phenolic moiety in comparison to the second phenol group amounts to ~0.03 Å, respectively.

Conformers III, IV, VII and VIII of the *E*-enol-imine tautomer are higher in energy than the most stable form by over 55 kJ mol^−1^, due to the breaking of the intramolecular O–H···N bond and its replacement by the repulsive interaction between the nitrogen and oxygen lone electron pairs. Among the high-energy conformers, forms XI, XII, XV and XVI are the less energetic (relative energies between 38 and 40 kJ mol^−1^; see Table 2) because they bear a C–H···O(H) attractive interaction between the CH group of the azomethine bridge and the phenolic oxygen atom (of the bromo-substituted phenolic ring), while conformers IX, X, XIII and XIV possess a C–H···H–O repulsive interaction and are intermediate in energy, with relative energies between 44 and 47 kJ mol^−1^. The C–C–O–H (δ) and C–C–C=N (ϒ) dihedral angles in forms IX, X, XIII and XIV clearly reveal the relevance of the C–H···H–O repulsive interaction: in these conformers, (i) the OH group is tilted from the plane of the aromatic ring (δ is in the range 6–9°), while it is deviated by less than 1° from the ring plane in all the other *E*-enol-imine conformers, and (ii) the dihedral angle ϒ, which is a measure of the coplanarity between the azomethine group and the bromo-substituted phenol ring, have values corresponding to geometries where the two groups deviate from by 7–9° from coplanarity, while for all the other *E*-enol-imine conformers the deviation is in the range of ~0–4° (see Table 1). 

In the case of the *Z*-enol-imine isomer, none of the sixteen distinct conformers have any strong intramolecular H-bond, this being one of the main reasons for their much higher relative energies (63–75 kJ mol^−1^) in comparison with the four lowest energy conformers of the *E*-enol-imine isomer (together with the already mentioned repulsions between the two phenolic rings). The most stable *Z*-enol-imine conformers (all with energies ~63 kJ mol^−1^ relatively to *E*-enol-imine V) are forms I, II, V and VI, and possess a weak O–H···*π* intramolecular interaction (see Figure 3 and Table 2), which justifies their lower energy compared with the remaining *Z*-enol-imine conformers. Conformers XI, XI, XV, and XVI are the next in order of growing energy (~67 kJ mol^−1^). Like the related conformers of *E*-enol-imine, these conformers bear a weak C–H···O(H) attractive interaction between the CH group of the azomethine bridge and the phenolic oxygen atom of the bromo-substituted phenol ring. Also as their analogues in *E*-enol-imine, conformers *Z*-enol-imine IX, X, XIII and XIV (~70 kJ mol^−1^) possess a C–H···H–O repulsive interaction, which likewise reflects in the values of the C–C–O–H (δ) and C–C–C=N (ϒ) dihedral angles that evidentiate the tilt of the OH group out of the plane of the aromatic ring (δ is in the range 7–10°) and the strong deviation from coplanarity (by 25–31°) of the azomethine and bromo-substituted phenol moieties (see Table 1). It is interesting to note that in the case of the *Z*-enol-imine conformers, the most out of the plane tilted OH group, as measured by the dihedral angle δ, are conformers I, II, V, and VI, due to the fact that in these conformers the OH group has to adapt its geometry to better fulfil the O–H···*π* stabilizing intramolecular interaction (see Figure 3). This fact also explains the strong non-coplanarity of the azomethine and bromo-substituted phenol moieties in these conformers, as measured by the dihedral angle ϒ (see Table 1). The highest energy *Z*-enol-imine conformers (73–75 kJ mol^−1^) are forms III, IV, VII, and VIII, mostly due to effect of the repulsive interaction between the lone electron pairs of the azomethine nitrogen and bromo-substituted phenolic oxygen atoms (see Figure 3). It is in these conformers that the dihedral angle ϒ assumes the values (~47°) that correlate with a maximum deviation from coplanarity of the azomethine and bromo-substituted phenolic fragments.

Considering only the *Z*-enol-imine conformers, the energies are within 12 kJ mol^−1^, i.e., they span a much narrower range of values compared to what happens for *E*-enol-imine. The main reason for this is that in *Z*-enol-imine no conformers exist that are stabilized by a strong intramolecular H-bond (if we do not count with those, the energies of the *E*-enol-imine conformers differ only by 19 kJ mol^−1^, nevertheless still a somewhat larger value compared to what is observed for *Z*-enol-imine). 

A final note shall be done regarding the geometries around the N=C double bond. For both *E*- and *Z*-enol-imine isomers, all conformers show the azomethine bridge slightly non-planar. The C–C=N–C dihedral stays in the range ~5–8° in the case of the *Z*-enol-imine conformers, where the repulsions between the two phenol moieties dominate, and between ~2–4° in the case of the *E*-enol-imine conformers. This structural feature has already been pointed out before for other aryl Schiff bases [26,28,29,30,47,48].

The keto-amine tautomer of BHAP can also exist in two geometrical isomeric species (*E* and *Z*) differing in the placement of the substituents around the C=C bond of the bridge. Since the geometric isomers of this tautomer do not have a direct structural correspondence to the geometric isomers of the enol-imine tautomer, in this article the keto-amine conformers of BHAP have been grouped in two sets, one structurally related to the *E*-enol-imine tautomer and the other to the *Z*-enol-imine tautomer, which will be designated as “keto-amine forms structurally related to the *E*-enol-imine tautomer” and “keto-amine forms structurally related to the *Z*-enol-imine tautomer”, respectively. In both sets, there are *Z*-keto-amine and *E*-keto-amine conformers. In the keto-amine forms, there are three conformationally relevant internal axes of rotation: one of these (α in Figure 4) corresponds to the rotation around the phenolic C–O bond and define the orientation of the phenolic hydrogen atom, while the others (β and ϒ in Figure 4) are associated with the C–N bonds of the bridge (the first connecting the N atom to the phenol ring, and the second being the central bond of the bridge), and determine the relative orientation of the two aromatic rings. The performed B3LYP/6-311++G(d,p) calculations predicted the existence of 8 distinct conformers for each keto-amine geometrical isomer, which have been grouped in Figure 4 according to their structural relation with the two enol-imine isomers, as stated above. The conformationally relevant dihedral angles of the keto-amine conformers are provided in Table 3, and the corresponding electronic energies (with and without zero-point correction), Gibbs standard energies (at 298.15 K), and dipole moments are given in Table 4.

The DFT performed calculations indicate that all keto-amine conformers structurally related to the *E*-enol-imino isomer have lower energies than those related with the *Z*-enol-imino isomer (see Table 4). Conformers I, II, V, and VI of the first group are considerably lower in energy (by over ~40 kJ mol^−1^, compared to the remaining *E*-enol-imino related keto-amine conformers, and by over 80 kJ mol^−1^, compared to all the *Z*-enol-imine related keto-amine conformers). This could be anticipated, since these conformers have a strongly stabilizing N–H···O=C intramolecular H-bond interaction.

The N–H···O=C H-bond interaction is indeed strong, as reflected in the H-bond structural parameters. The N–H bond length in all the keto-amine conformers having this interaction is 1.042 Å, and the H···O distance stays in the range 1.682–1.700 Å, which is considerably shorter than the sum of H+O van der Walls radii (1.20 + 1.52 = 2.72 Å). The O···N distance is in the 2.579–2.588 Å range, being shorter (as the H···O distance) in conformer V, and longer in conformer II, while the N–H···O angles are ~140° in all the four conformers.

As seen in Table 3, conformers I, II, V and VI are almost planar, whereas conformers III, IV, VII and VIII are considerably non-planar, exhibiting a dihedral angle β equal to 166.5, 169.9, −8.7, and −9.0°, respectively, because of the repulsive interaction between the hydrogen linked to the carbon atom of the bridge and the closely located hydrogen of the phenol group (see Figure 4). All the keto-amino conformers related to the *Z*-enol-imine isomer are strongly non-planar due to the close proximity of the two phenolic moieties.

Looking now to the energy differences within the conformers of the *E*-keto-amine and *Z*-keto-amine forms (these values are given in the last columns of Table 4 and distinguished using italic type and round type respectively), one can see that in each case the four conformers related to the *E*-enol-imine form are considerably more stable than those related to the *Z*-enol-imine isomer. In the case of the *E*-keto-amine isomer, the energies of the conformers span through a range of ~37 kJ mol^−1^, the four most stable conformers being within a ~3 kJ mol^−1^ energy range. For the *Z*-keto-amino isomer, the energies of the conformers span for a range of ~66 kJ mol^−1^, the four most stable conformers being again within a ~3 kJ mol^−1^ energy range.

A general conclusion that can be extracted from the theoretical calculations is that, when the *E*- and *Z*- isomeric forms of the two tautomers (enol-imine and keto-amine) are considered as independent species, the gas phase equilibrium populations of the corresponding most stable four conformers shall be roughly equal to ~25% each. As described below, this is an important piece of information for the analysis of the obtained matrix isolation experimental data.

### 3.2. Natural Bond Orbital Analysis

In order to look into more detail to the electronic structure characteristic of BHAP, natural bond orbital (NBO) analysis [49] was performed using the DFT/B3LYP/6-311++G(d,p) data. The lowest energy conformer *E*-enol-imine V was used as target for these calculations.

The calculated NBO atomic charges are listed in Table 5. As expected, the NBO atomic charges show that the hydrogen-bonded phenolic OH group (O15–H26, according to the adopted atom numbering scheme, shown in the insert of Table 5) is considerably more polarized than the free phenolic OH group (O17–H27), due to the involvement of the first in the hydrogen bond: the positive charge of the H-bonded H27 atom is considerably larger than that of H26 (+0.507 vs. +0.468 *e*), but the negative charge of O15 is also slightly more negative than that of O17 (–0.677 vs. –0.670 *e*). 

The charges of the aromatic hydrogen atoms vary in the range +0.207 to +0.224 *e*, the more positive ones belonging to the hydrogen atoms of the bromo-substituted phenolic ring, while that of the hydrogen atom of the azomethinic bridging group is +0.170 *e*. The aromatic carbon atoms have calculated negative charges within the range −0.171 to −0.278 *e*, except that connected to the Br atom, which has a less negative charge (−0.138 *e*), and those connected to the oxygen and nitrogen atoms, which are positive. As expected, the carbon atom connected to the more negative O15 atom has a larger positive charge (+0.376 *e*) than that connected to O17 (+0.325 *e*), while the carbon atoms linked to the nitrogen atom (whose charge is −0.522 *e*, i.e., less negative than those of the oxygen atoms) have positive charges, +0.154 *e* (C5) and +0.162 *e* (C8). The Br atom is predicted to have a slightly positive charge, pointing to a more pronounced π-donation to the ring, compared to the σ-withdrawn.

The nature of the NBOs (composition in terms of atomic orbitals) provides an elegant way to define the hybridization adopted by the different atoms in the molecule. Table 6 shows the description of the occupied bonding NBOs and those associated with lone electron pairs in terms of the atomic contributions and the corresponding occupancies. The descriptions allow to extract information about the hybridization assumed by the different atoms in the molecule.

All carbon atoms except C1 exhibit the expected hybridization pattern, using a p orbital to form the π component of the double bonds, and typical sp^2^ hybrid orbitals to form the σ bonds (including the σ component of the double bonds). In all cases, the sp^2^ hybrids used to establish a bond with a hydrogen atom have slightly larger p character, while those used to form the σ bonds with other carbon atoms have a larger s contribution. The p contribution to the hybrid orbitals are large when the carbon is connected to the more electronegative Br, N, and O atoms, the extreme case corresponding to the bond between C1 and O17, where the carbon atom uses an essentially pure p orbital. The orbital mixing calculated for C1 is in fact unusual, since it uses a p orbital to establish the linkage with O17, two hybrids orbitals with substantial s character to form the σ bonds with the vicinal ring carbon atoms (C2 and C6) and an orbital with large p character, but still significant s contribution, to form the π bond in which it is involved. The pattern of hybridization adopted by the two oxygen atoms is also interesting, though both can be considered to be formally hybridized sp^2^. O15 has its lone pairs occupying a p and a sp^2^ hybrid orbital (the latter with an increased s character), the two additional hybrid orbitals being used to form the bonds with C14 and H26, the last one having an increased p character (approaching the typical p composition of a sp^3^ hybrid orbital). In turn, O17 has its lone pairs occupying sp^2^ hybrid orbitals, one of them with a large s character, uses the third hybrid orbital, which has a large p character, to establish the bond with the hydrogen atom, and binds to the ring carbon atom (C1) using a p orbital. The difference revealed by the NBO analysis in the electronic structures of the two oxygen atoms is most probably a result of the involvement of O15 in the strong intramolecular O–H···N hydrogen bond, which also has implications on the electronic structure around the N atom. Indeed, the N atom has a hybridization rather close to sp, with two essentially pure p orbitals being used to accommodate the lone pair and form the π bond with C8, and the two hybrid orbitals to form the σ bonds with C5 and C8.

Finally, the bromine atom is not hybridized, using one of its p orbitals to form the bond with C11, while the three lone pairs occupy the s and the remaining two p valence orbitals.

The bond orders (BO) were evaluated using the natural atomic orbitals Wiberg bond index [50]. The results are given in Table 7. The bond orders for the carbon-carbon and C–H bonds of the aromatic rings are in the range of 1.27–1.47 and equal to 0.92, respectively. In the case of the carbon-carbon bonds, the bonds with the smallest bond orders in the bromo-substituted phenol ring are the C9–C14, C9–C10, and C13–C14 bonds, while those with the largest bond orders are the C12–C13 and C10–C11 bonds. The first involve the carbon atoms bearing the OH and azomethine substituents, and participate in the 6-membered pseudo-ring containing the intramolecular hydrogen-bond; as expected these bonds are the longest bonds in the bromo-substituted phenol ring (1.419, 1407, and 1.400 Å, respectively). In turn, the largest bond order bonds are vicinal to the previous ones and the shortest bonds in the ring (1.386 and 1.381 Å, respectively).

A similar trend is observed for the second phenol ring, with the bonds involving C5 and C1, which bear the substituents, having the smallest bond orders and being the longest bonds. The bond order of the exocyclic C8–C9 bond is somewhat larger than the unity (1.14), expressing the π delocalization between the bromo-substituted phenol ring and the azomethine bridge, which are co-planar. This is also reflected in the calculated bond order for the central N7=C8 bond (1.70), which is smaller than the formal value for a non-delocalized double bond. In turn, the bond order of the C5–N7 bond is close to 1, since the phenol ring and the azomethine group are considerably tilted in relation to each other (see Table 1 and discussion on Section 3.1) and delocalization is then precluded. The two C–O bonds have bond orders close to the unity, but that of the C1–O17 bond is somewhat higher than the one of the C14–O15 bond, in consonance with their relative lengths (1.367 vs. 1.340 Å). On the other hand, the bond orders and corresponding bond lengths of the two O–H bonds are very much different, in result of the participation of the O15–H26 group in the intramolecular hydrogen bond. While for this group the bond order is only 0.64 and the bond length is 1.993 Å, for the O17–H27 group the bond order is 0.77 and the bond length is 1.963 Å. The calculated bond order for the N···H hydrogen bond is 0.09, which is a value similar to those found for other molecules bearing a strong intramolecular hydrogen bond [51].

### 3.3. Infrared Spectrum of BHAP in an Ar Matrix

As described in the Section 2.2 vapors of BHAP were co-deposited with a large excess of argon onto a cold (10 K) CsI substrate mounted at the cold tip of the cryostat. The purposes of this experiment were: (i) to verify the nature of the species trapped in the matrix; (ii) to perform the vibrational study of these species. A few considerations shall be made *a priori*. Firstly, the four isomeric species discussed in the previous sections (*E*- and *Z*-enol-imine and *E*- and *Z*-keto-amine isomers) cannot interconvert in the gas phase nor in the matrices, since the energy barriers associated with these processes are very high (over a few hundreds of kJ mol^−1^ [52], because they imply breaking of a double bond, N=C or C=C). Secondly, the IR data obtained for the crystalline material in a KBr (room temperature; Supplementary Figure S1) indicate that in the crystal an enol-imino form is present, since no band ascribable to the stretching vibration of the carbonyl group of the keto-amine forms is observed. Because the *E*-enol-imine isomer is considerably more stable that the *Z*-enol-imine one, the first form could be expected to be the one resulting from the synthesis. Nevertheless, this hypothesis should be verified. Finally, as already mentioned, the conformational population in the gas phase equilibrium, after sublimation of the compound, shall comprehend conformers I, II, V, and VI in nearly equal amounts. This applies for the *E*-enol-imine isomer, but also for the *Z*-enol-imine isomer in the case this higher energy species reveals to be experimentally relevant.

**Figure 5:**
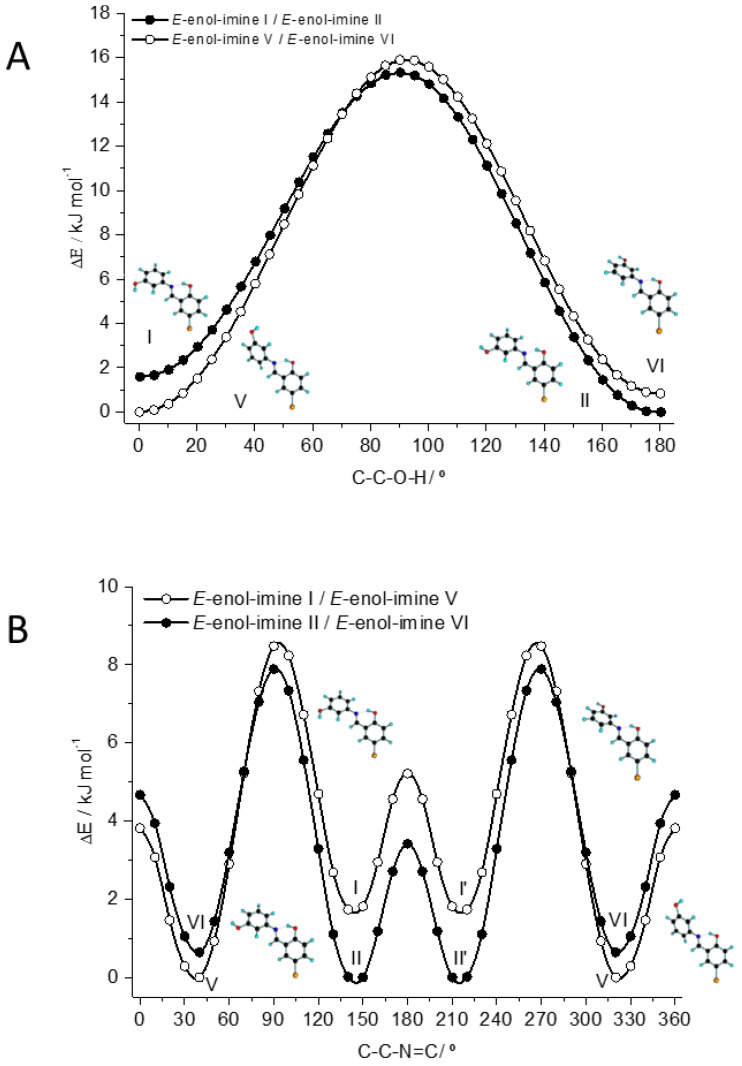
Potential energy profiles for interconversion between the four most stable conformers of the *E*-enol-imine isomer by internal rotation around the C–O bond (**A**, top panel) and C5–N bond (**B**, bottom panel). In B, I/I’ and II/II’ represent pairs of symmetry-equivalent conformers.

It is also worth mentioning that, in a matrix isolation experiment, higher-energy conformers may convert into lower energy forms during deposition (conformational cooling effect), if the energy barriers separating the conformers are small enough (a few kJ mol^−1^) [33,34,53,54,55,56]. In addition, moderate size barriers (up to ca. 20–25 kJ mol^−1^) associated with transformations involving only movement of a hydrogen atom may also allow conformational decay by quantum mechanical tunneling through the barrier, this phenomenon being common for molecules bearing the hydroxyl moiety, in particular for phenols and naphtols and for carboxylic acids [57,58,59,60,61,62]. In order to estimate the energy barriers separating the low-energy conformers of BHAP, relaxed potential energy scans were performed (at the B3LYP/6-311++G(d,p) level) along the relevant coordinates. In the studied molecule, the four low-energy *E*-enol-imine conformers (I, II, V and VI) can be interconverted by two ways: (i) by rotation of the phenolic OH around the C–O bond; and (ii) by rotation of the whole phenol group around the C–N bond connecting to phenol and azomethine fragments. The corresponding potential energy scan profiles are shown in Figure 5.

The conformational isomerization energy barriers associated with the internal rotation around the C5–N bond are ~7–9 kJ mol^−1^ (Figure 5B). These barriers are not large, but still large enough to preclude the conformational cooling to take place during deposition (at least in a significant way) [53]. So, taking into account only this conformational path, in principle, all 4 low-energy conformers should be trapped in the cryogenic matrix. On the other hand, the internal rotation of the OH group implies to overcome a barrier of ~15 kJ mol^−1^ (Figure 5A). This barrier is enough high to preclude the OH group to rotate at the cryogenic temperature of the matrix in an over-the-barrier process. However, as mentioned above, hydrogen tunneling through a barrier of such height is highly probable, so that conformers I and VI can be expected to quickly convert into conformers II and V, respectively. This has been observed to happen in phenols and other similar compounds [59,60]. As a whole, the calculated data on the energy barriers then support the presence of only conformers II and V of the *E*-enol-imine isomer in the matrix in nearly equal amounts.

**Figure 6:**
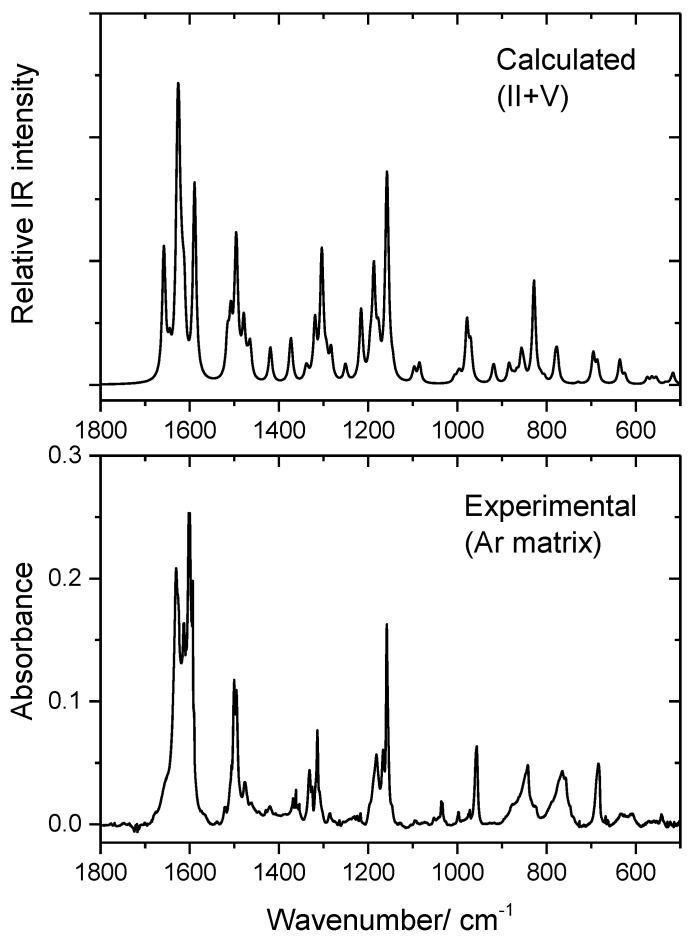
Infrared spectrum of BHAP in an Ar matrix (10 K) in the 1800–500 cm^−1^ range (*bottom*), and sum spectrum of the B3LYP/6-311++G(d,p) calculated spectra for *E*-enol-imine conformers II and V.

Figure 6 shows the infrared spectrum of BHAP isolated in an argon matrix, at 10 K. In the figure, the sum spectrum of the B3LYP/6-311++G(d,p) calculated spectra for *E*-enol-imine conformers II and V is also presented. Figure S4, in the Supporting Information, shows also the sum spectra of the B3LYP/6-311++G(d,p) calculated infrared spectra for *Z*-enol-imine conformers II and V, and also those for *E*- and *Z*-keto-amine isomers built based on the calculated spectra of their hypothetically experimentally relevant conformers, for comparison.

As it can be seen in Figure 6 and Supplementary Figure S4, the data clearly shows that in the matrix only the *E*-enol-imine isomer is present, the spectra of the remaining isomeric species strongly differing from the experimental spectrum. The good reproduction of the experimental spectrum by the simulated one based on conformers II and V of the *E*-enol-imino isomer indicated also that the considerations above were most probably correct. However, because the infrared spectra of the four low-energy conformers of the *E*-enol-imine isomer do not differ significantly, it is not possible, based only on the comparison of the experimental and simulated spectra, to conclude with all certainty that conformers I and VI are not also contributing to the observed spectrum. Nevertheless, while the frequencies are very similar all among the four conformers, intensities are somehow different in several spectral regions. Thus, annealing of the matrix should, in principle, allow for isomerization of the putative conformers I and VI into conformers V and II, respectively, through internal rotation around the C5–N bond (see Figure 5B). Nonetheless, annealing of the matrix up to 25 K did not lead to any spectral changes, strongly supporting the sole presence of conformers II and V in the matrix.

The molecule of BHAP has 27 atoms, i.e., 75 fundamental vibrations, all active in IR, with 47 modes predicted by the calculations to appear in the 1800–500 cm^−1^ region. The proposed assignments are presented in Table 8. Below, some of the assignments are discussed briefly.

Above 1800 cm^−1^, only the OH and CH stretching vibrations are expected to absorb. The aromatic CH stretchings were found to span the ~3100–2900 cm^−1^ range, while the azomethine CH stretching mode is observed as a pair of bands at 2864 and 2846 cm^−1^, which are tentatively assigned to conformers V (calc. 2878 cm^−1^) and II (calc. 2877 cm^−1^), respectively. The stretching mode of the free OH group is assigned to the pair of bands at 3642 (II; calc. 2625 cm^−1^) and 3640 cm^−1^ (V; calc. 3623 cm^−1^), while the intramolecularly hydrogen bonded OH group gives rise to a broad and structured feature spreading over the very wide 3573–3035 cm^−1^ region, as it is normally observed for hydrogen bonded hydroxyl groups [63].

In the 1800–500 cm^−1^ spectral region, the experimental spectrum shows intense bands in the 1650–1590 and 1200–1150 cm^−1^ ranges, and around 1500, 1320, 960, 840, 760 and 680 cm^−1^. This is in full agreement with the results of the calculations, as can be seen in Figure 6 and also in Table 8. 

The 1650–1590 cm^−1^ region comprehends the bands due to the νN=C stretching mode (exp.: 1630 and 1626 cm^−1^; calc.: II, 1658 cm^−1^ and II, 1656 cm^−1^) and the highest frequency νCC stretching modes of the aromatic rings, some of them significantly mixed with the bending vibration (δOH) of the corresponding phenolic OH group. Indeed, according to the calculations, the intense band observed at 1593 cm^−1^ corresponds to the mode with a larger contribution of the bending mode of the H-bonded phenolic OH group. As expected, this mode appears considerably shifted to higher frequencies when compared to the equivalent vibration of the free OH group, which is assigned to the bands at 1198 and 1181 cm^−1^, in conformer II (calc: 1195 cm^−;1^) and V (calc: 1187 cm^−1^), respectively. 

The group of observed bands around 1500 cm^−1^ correspond essentially to mixed ring νCC stretching and δCH bending modes, in some cases also mixed with the δOH bending modes, which contribute to the intensification of the bands. The δCH bending vibration of the azomethine group is assigned to the experimental bands at 1368 and 1358 cm^−1^, tentatively ascribed to conformer II and V, respectively (calc: 1374 and 1372 cm^−1^).

Among the vibrations contributing to the bands observed in the experimental spectrum at around 1320 cm^−1^, the νCO stretching modes of the phenolic groups shall be highlighted. As expected, the higher polarized (but with a higher bond order) C–O bond of the H-bonded phenolic group gives rise to a considerably more intense band (observed at 1314 cm^−1^) than the C–O bond of the free phenolic group (observed at 1308 cm^−1^ for V and 1285 cm^−1^ for II).

In the 1200–1150 cm^−1^ range, besides the bands assigned to the δOH bending mode of the free phenol OH group in the two conformers, another intense band is observed at 1158 cm^−1^, which, according to the calculations, is ascribable to a mixed δCH/νCO/νC–N mode (Table 8).

Finally, the intense experimental bands at ca. 960, 840, 760, and 680 cm^−1^ are due to a ring δCC/δCH deformation mode with a relevant contribution from the νCO streching of the free phenolic moiety, the ϒOH torsion of the hydrogen bonded OH group, the ring all-in-phase out-of-the-plane ϒCH rocking modes, and ring ϒCC torsional modes, respectively. As expected [64], the ϒOH torsion of the hydrogen bonded OH group appears at a much higher frequency than the equivalent mode in the free OH group, which is predicted by the calculations at ~320 cm^−1^. 

## 4. Conclusions

In this study, a detailed structural investigation of a new *o*-hydroxyaryl Schiff base was undertaken. Quantum chemical electronic structure calculations, performed at the DFT(B3LYP)/6-311++G(d,p) level, were used to study the conformational space of the two geometrical isomers (*E* and *Z*) of the enol-imine and keto-amine tautomers of the compound. These studies allowed to conclude that the most stable isomeric form is the *E*-enol-imino form, and that this form has four low-energy, intramolecularly H-bonded conformers which are nearly degenerate.

The infrared spectrum of the crystalline material (in a KBr pellet at room temperature) showed that the synthesized solid material was an enol-imino form of the compound, a result that was also in consonance with the ^1^H- and ^13^C-NMR spectroscopy data obtained in DMSO-d_6_ solution. In turn, the low temperature matrix isolation infrared spectra demonstrated that the matrix isolated molecules, trapped from the room temperature Schiff base vapor, correspond to the *E*-enol-imine isomer in two specific conformers, II and V, which exist in the matrix in nearly equal amounts. The conjugation of these experimental results with simple chemical considerations on the structural relations between the enol-imine and keto-amine isomers permitted to conclude that the synthesized crystalline material corresponds to the *E*-enol-imine tautomer of the studied compound, which upon sublimation gives rise to a vapor where the four low-energy conformers (I, II, V and VI) bearing a strong O–H···N intramolecular hydrogen bond coexist with identical populations in the equilibrium. Deposition of the vapor of the compound with a large excess of argon onto a substrate kept at 10 K gives rise to a solid argon matrix of the compound, where only conformers II and V subsist, conformers I and VI spontaneously converting into forms II and V, respectively, by the quantum mechanical tunneling mechanism. These conformational decays, involve the conformational change of the free OH phenolic group and takes place by hydrogen atom tunneling through a barrier for internal rotation about the C–O bond that is of ca. 15 kJ mol^−1^. 

Additional details of the electronic structure of the experimentally relevant isomeric species were obtained using the natural bond orbital (NBO) theory. This analysis targeted the most stable conformer of the *E*-enol-imino isomer (conformer V), and allowed to get insights on the hybridization states of the different atoms in the molecule, and their charges, as well as on the polarization and bond orders of the different bonds in the molecule, including the intramolecular hydrogen-bond. From all the different analyses performed, the structural relevance of the O–H···N intramolecular H-bond in the studied *o*-hydroxyaryl Schiff base was highlighted. Additionally, the matrix isolation infrared spectrum of the studied compound was fully assigned and interpreted with help of the DFT(B3LYP)6-311++G(d,p) calculated infrared spectra for the *E*-enol-imine conformers II and V.

## Figures and Tables

**Table 1:** B3LYP/6-311++G(d,p) optimized conformationally-relevant dihedral angles of the *E*-enol-imine and *Z*-enol-imine conformers of BHAP *^a^*.

		Dihedral Angles			
Conformer	C–C=N–C	C–C–O–H α	C–C–N=C β	C–C–C=N ϒ	C–C–O–H δ
*E*-enol-imine					
I	−177.2	0.3	37.8	0.7	−0.3
II	−177.3	178.6	36.9	0.6	−0.3
III	−177.4	0.5	43.5	4.4	179.3
IV	−177.4	179.1	43.2	4.3	179.1
V	−177.1	0.2	−144.5	0.6	−0.1
VI	−177.1	−179.1	−143.6	0.6	−0.3
VII	−177.0	0.2	−140.9	2.7	178.7
VIII	−177.2	−178.3	−139.2	2.5	179.4
IX	−175.6	1.6	42.0	−171.4	9.4
X	−176.2	178.9	40.6	−173.4	5.6
XI	−177.3	0.2	39.6	−178.5	178.7
XII	−177.3	179.2	39.6	−178.4	179.8
XIII	−175.9	−0.8	−141.9	−172.6	6.4
XIV	−176.0	−178.5	−140.4	−173.0	6.8
XV	−177.1	0.1	−144.1	−178.9	179.6
XVI	−177.2	−178.8	−142.3	−179.3	179.4
*Z*-enol-imine					
I	7.2	0.6	−128.6	38.8	−23.5
II	6.7	−179.5	−126.9	39.1	−21.1
III	5.5	−1.4	−133.1	47.2	−175.0
IV	5.7	−176.3	−131.9	47.1	−176.5
V	6.1	−0.5	58.2	39.5	−21.9
VI	6.3	−179.4	58.6	38.9	−21.7
VII	5.1	0.9	52.7	47.3	−175.4
VIII	5.3	−178.2	53.2	46.5	−176.0
IX	7.8	0.8	−127.1	−148.7	8.2
X	7.3	−179.4	−124.7	−148.5	7.8
XI	8.1	0.5	−122.9	−154.6	−176.9
XII	7.6	−179.2	−120.7	−154.4	−176.6
XIII	7.2	−1.5	59.3	−148.4	6.6
XIV	7.3	−178.7	60.9	−148.7	9.3
XV	7.3	−0.2	65.2	−154.5	−176.0
XVI	7.4	−179.2	64.7	−153.8	−177.0

*^a^* Angles in degrees; for definition of dihedral angles and structures of the conformers, see Figure 2 and Figure 3. Note that all conformers have a symmetry related structure.

**Table 2:** B3LYP/6-311++G(d,p) calculated relative electronic energies (ΔE_el_), zero-point corrected relative energies (ΔE_(0)_), standard Gibbs energies at 298.15 K (ΔG°_298.15_), and dipole moments (μ) for the enol-imine conformers of BHAP *^a^.*

Conformer	ΔE_el_	ΔE_(0)_	ΔG°_298.15_	μ	ΔE_el_	ΔE_(0)_	ΔG_298.15_
*E*-enol-imine							
I	1.69	1.51	0.70	1.36			
II	0.10	0.15	−0.51	3.35			
III	56.77	54.10	51.30	2.78			
IV	55.71	53.20	50.51	1.63			
V	0.00	0.00	0.00	1.01			
VI	0.84	0.67	0.67	3.53			
VII	54.95	52.51	49.66	2.95			
VIII	56.38	53.65	50.51	0.91			
IX	47.18	44.45	41.31	2.43			
X	45.47	42.88	38.97	3.33			
XI	39.76	37.18	34.54	4.57			
XII	38.89	36.58	34.25	3.20			
XIII	44.90	42.38	39.57	1.83			
XIV	46.28	43.51	40.72	3.88			
XV	38.13	35.68	32.73	3.89			
XVI	39.62	36.86	33.64	4.33			
*Z*-enol-imine							
I	62.99	61.92	61.52	0.80	0.25	0.29	0.46
II	63.25	61.94	61.44	2.72	0.51	0.31	0.37
III	73.30	71.33	70.23	2.93	10.56	9.70	9.16
IV	75.05	72.47	70.54	3.88	12.31	10.84	9.48
V	62.74	61.63	61.07	0.51	0.00	0.00	0.00
VI	63.12	61.73	60.93	2.80	0.38	0.10	−0.14
VII	74.60	72.25	70.38	2.96	11.86	10.62	9.31
VIII	75.01	72.47	70.53	3.97	12.27	10.84	9.46
IX	70.42	68.65	66.04	1.66	7.68	7.02	4.98
X	70.67	68.82	66.26	1.82	7.93	7.19	5.20
XI	66.67	64.98	62.18	4.31	3.93	3.35	1.11
XII	67.09	65.26	62.41	3.92	4.35	3.63	1.34
XIII	71.27	69.42	66.59	2.17	8.53	7.79	5.52
XIV	70.42	68.58	65.80	0.72	7.68	6.95	4.73
XV	67.35	65.73	62.82	4.88	4.61	4.10	1.76
XVI	66.87	65.14	62.33	2.89	4.13	3.51	1.26

*^a^* Energies in kJ mol^−1^; dipole moments in Debye. See Figure 2 and Figure 3 for the structures of the conformers. In the case of the conformers of *Z*-enol-imine, the first set of energy values are relative to the most stable form, *E*-enol-imine V, and the second set are relative to the most stable conformer of the *Z*-enol-imine isomer.

**Table 3:** B3LYP/6-311++G(d,p) optimized conformationally-relevant dihedral angles of the keto-amine forms of the *E*- and *Z*-enol-imine isomers of BHAP *^a^*.

	Dihedral Angles			
Conformer	C–C=C–N	C–C–O–H α	C–C–N–C β	C–N–C=C ϒ
keto-amine forms structurally related to the *E*-enol-imine isomer				
I	0.0	0.0	0.0	179.9
II	0.0	−179.9	−0.7	179.9
III	178.4	0.6	166.5	177.5
IV	179.0	−179.3	169.9	178.4
V	0.0	0.4	175.1	179.6
VI	0.0	−179.6	174.1	179.0
VII	179.1	1.6	−8.7	178.6
VIII	178.7	−179.0	−9.0	178.6
keto-amine forms structurally related to the *Z*-enol-imine isomer				
I	176.0	4.7	−44.8	−16.3
II	177.1	179.5	−47.3	−15.7
III	−14.9	−3.7	153.1	−24.5
IV	−15.3	−176.6	156.0	−23.9
V	177.4	−1.2	131.6	−15.1
VI	176.6	−177.9	136.9	−16.1
VII	−15.6	8.8	−25.6	−23.3
VIII	−15.3	178.3	−28.2	−24.2

*^a^* Angles in degrees; for definition of dihedral angles and structures of the conformers, see Figure 4. Note that all conformers have a symmetry related structure.

**Table 4:** B3LYP/6-311++G(d,p) calculated relative electronic energies (ΔE_el_), zero-point corrected relative energies (ΔE_(0)_), standard Gibbs energies at 298.15 K (ΔG°_298.15_), and dipole moments (μ) for the keto-amine conformers of BHAP *^a.^*

Conformer	ΔE_el_	ΔE_(0)_	ΔG°_298.15_	μ		ΔE_el_	ΔE_(0)_	ΔG_298.15_
keto-amine forms structurally related to the *E*-enol-imine isomer								
I	19.69	18.94	16.35	3.49		2.75	2.66	2.39
II	16.95	16.28	13.96	5.30		0.00	0.00	0.00
III	61.49	61.03	56.80	4.74		*2.95*	*2.86*	*3.82*
IV	58.54	58.17	52.98	5.67		*0.00*	*0.00*	*0.00*
V	17.87	17.01	11.28	2.97		0.92	0.72	–2.68
VI	18.42	17.67	14.20	5.67		1.48	1.39	0.25
VII	58.66	58.14	52.81	3.82		*0.12*	*–0.03*	*–0.17*
VIII	59.08	58.45	52.93	6.41		*0.54*	*0.28*	*–0.05*
keto-amine forms structurally related to the *Z*-enol-imine isomer								
I	80.86	81.47	78.49	4.46		63.91	65.19	64.53
II	82.00	82.43	79.19	6.89		65.05	66.15	65.23
III	94.97	94.96	93.91	4.53		*36.43*	*36.79*	*40.93*
IV	92.80	92.95	92.27	4.05		*34.26*	*34.78*	*39.29*
V	82.94	83.08	79.48	5.39		65.99	66.80	65.52
VI	81.05	81.44	78.61	6.29		64.09	65.16	64.66
VII	91.88	92.47	92.00	3.91		*33.34*	*34.30*	*39.02*
VIII	93.01	93.29	92.87	4.75		*34.47*	*35.12*	*39.89*

*^a^* Energies in kJ mol^−1^; dipole moments in Debye. See Figure 4 for the structures of the conformers. The first set of energy values is relative to the most stable form, *E*-enol-imine V, and the second set is relative to the most stable conformer of each keto-amine isomer. Values in *italic* style are for *E*-keto-amine forms and values in round style for *E*-keto-amine forms.

**Table 5:** DFT(B3LYP)/6-311++G(d,p) NBO atomic charges (in units of electron; *e* = 1.60217646 × 10^−19^ C) for the *E*-enol-imine V conformer.

Atom	NBO Charge	Atom	NBO Charge	
C1	0.325	O15	−0.677	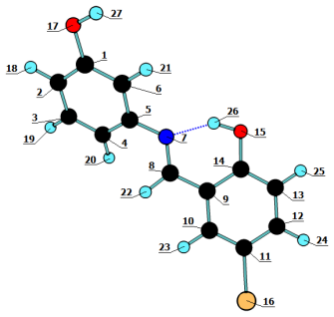
C2	−0.253	Br16	0.061	
C3	−0.171	O17	−0.670	
C4	−0.256	H18	0.220	
C5	0.154	H19	0.207	
C6	−0.278	H20	0.209	
N7	−0.522	H21	0.209	
C8	0.162	H22	0.170	
C9	−0.176	H23	0.219	
C10	−0.175	H24	0.223	
C11	−0.138	H25	0.224	
C12	−0.182	H26	0.507	
C13	−0.238	H27	0.468	
C14	0.376			

**Table 6:** Selected (bonding and lone pair occupied) NBOs for *E*-enol-imine V conformer obtained from the DFT/B3LYP/6-311++G(d,p) calculations.

		Coefficients (%) *^b^*		
Orbital	Occupancy (*e*) *^a^*	A	B	Description
σ(C1–C2)	1.97297	50.75	49.25	C1 sp^1.68^ + C2 sp^1.92^
π(C1–C2)	1.68279	49.24	50.76	C1 sp^3.35^ + C2 p
σ(C1–C6)	1.97708	50.10	49.90	C1 sp^1.60^ + C6 sp^1.85^
σ(C1–O17)	1.99029	11.14	88.86	C1 p + O17 p
σ(C2–C3)	1.90762	49.96	50.04	C2 sp^1.89^ + C3 sp^1.79^
σ(C2–H18)	1.92993	60.78	39.22	C2 sp^2.24^ + H18 s
σ(C3–C4)	1.97617	49.69	50.31	C3 sp^1.78^ + C4 sp^1.77^
π(C3–C4)	1.68051	46.96	53.04	C3 p + C4 p
σ(C3–H19)	1.97978	60.44	39.56	C3 sp^2.54^ + H19 s
σ(C4–C5)	1.97155	49.10	50.90	C4 sp^1.87^ + C5 sp^1.65^
σ(C4–H20)	1.97822	60.54	39.46	C4 sp^2.44^ + H20 s
σ(C5–C6)	1.96869	50.49	49.51	C5 sp^1.88^ + C6 sp^1.78^
π(C5–C6)	1.66431	46.58	53.42	C5 p + C6 p
σ(C5–N7)	1.98468	39.31	60.69	C5 sp^2.65^ + N7 sp^1.30^
σ(C6–H21)	1.97686	60.52	39.48	C6 sp^2.45^ + H21 s
σ(N7–C8)	1.98676	59.95	40.05	N7 sp^1.33^ + C8 sp^2.02^
π(N7–C8)	1.92978	70.93	29.07	N7 p + C8 p
σ(C8–C9)	1.97187	48.56	51.44	C8 sp^1.82^ + C9 sp^2.11^
σ(C8–H22)	1.97565	59.42	40.58	C8 sp^2.16^ + H22 s
σ(C9–C10)	1.96315	51.13	48.87	C9 sp^1.88^ + C10 sp^1.86^
π(C9–C10)	1.64114	54.94	45.06	C9 p + C10 p
σ(C9–C14)	1.97181	51.09	48.91	C9 sp^2.02^ + C14 sp^1.71^
σ(C10–C11)	1.97983	50.21	49.79	C10 sp^1.74^ + C11 sp^1.55^
σ(C10–H23)	1.97719	61.05	38.95	C10 sp^2.49^ + H23 s
σ(C11–C12)	1.97972	50.32	49.68	C11 sp^1.59^ + C12 sp^1.82^
π(C11–C12)	1.64989	56.19	43.81	C11 p + C12 p
σ(C11–Br16)	1.98466	49.53	50.47	C11 sp^3.51^ + Br16 p
σ(C12–C13)	1.96871	50.19	49.81	C12 sp^1.79^ + C13 sp^1.74^
σ(C12–H24)	1.97873	61.21	38.79	C12 sp^2.47^ + H24 s
σ(C13–C14)	1.97688	49.33	50.67	C13 sp^1.91^ + C14 sp^1.71^
π(C13–C14)	1.58461	54.83	45.17	C13 p + C14 p
σ(C13–H25)	1.97697	61.21	38.79	C13 sp^2.43^ + H25 s
σ(C14–O15)	1.99406	33.91	66.09	C14 sp^2.86^ + O15 sp^1.95^
σ(O15–H26)	1.98451	77.97	22.03	O15 sp^2.91^ + H26 s
σ(O17–H27)	1.98736	73.72	26.28	O17 sp^3.76^ + H27 s
Lp N7	1.60068			p
Lp1 O15	1.97390			sp^1.46^
Lp2 O15	1.81108			p
Lp1 Br16	1.99361			s
Lp2 Br16	1.97619			p
Lp3 Br16	1.94629			p
Lp1 O17	1.97920			sp^1.24^
Lp2 O17	1.35923			sp^2.01^

*^a^* Occupancy is given with an exaggerated accuracy, as in the Gaussian output file. *^b^* The A and B values correspond to the contributions of the atomic orbitals of the two atoms forming a bond, by the order indicated in the first column. Numbering of atoms as in the in-set figure shown in Table 5.

**Table 7:** Calculated bond orders, according to the Wiberg bond index *^a^*.

Bond	BO	Bond	BO	Bond	BO	Bond	BO
C1–C2	1.35	O17–H27	0.77	C8–C9	1.14	C14–C9	1.27
C2–C3	1.45	C2–H18	0.92	C8–H22	0.92	C14–O15	1.11
C3–C4	1.43	C3–H19	0.92	C9–C10	1.33	O15–H26	0.64
C4–C5	1.36	C4–H20	0.92	C10–C11	1.45	C10–H23	0.92
C5–C6	1.36	C6–H21	0.92	C11–C12	1.35	C11–Br16	1.03
C6–C1	1.39	C5–N7	1.07	C12–C13	1.47	C12–H24	0.92
C1–O17	1.02	N7–C8	1.70	C13–C14	1.34	C13–H25	0.92

*^a^* Numbering of atoms as in the in-set figure shown in Table 5.

**Table 8:** Assignment of the vibrational spectra of BHAP isolated in an argon matrix (10 K), and the B3LYP/6-311++G(d,p) calculated spectra for *E*-enol-imine conformers V and II *^a^*.

Exp.	Calculated			Calculated		
Ar Matrix *^b^*	V		Approximate Description *^c^*	II		Approximate Description*^c^*
ν	ν	I^IR^		ν	I^IR^	
*3642*/3640	3622.9	66.48	νOH_p_	3625.1	84.3	νOH_p_
3573–3035	3033.1	536.05	νOH_bp_	3033.6	538.1	νOH_bp_
3021	3027.7	2.81	νCH_bp_	3028.4	4.2	νCH_bp_
	3025.6	11.12	νCH_p_	3023.9	5.0	νCH_p_
	3015.9	4.44	νCH_p_	3019.4	2.0	νCH_p_
	3014.4	4.46	νCH_bp_	3014.8	4.0	νCH_bp_
3005	3008.2	1.29	νCH_bp_	3009.9	1.4	νCH_bp_
2993/*3005*	2998.2	8.52	νCH_p_	3005.8	17.9	νCH_p_
2987/*2975*	2991.1	7.78	νCH_p_	2983.9	10.3	νCH_p_
2864/*2846*	2878.3	34.13	νCH_am_	2877.4	34.5	νCH_am_
*1630*/1626	1656.5	97.93	νN=C; νCC_am_; δCH_am_	1658.1	117.5	νN=C; νCC_am_; δCH_am_
1613	1644.6	23.95	νCC_bp_; δOH_bp_	1644.7	14.5	νCC_bp_; δOH_bp_
1602/1600	1623.9	272.72	νCC_p_	1627.3	245.1	νCC_p_
	1617.6	80.99	νCC_p_; δOH_p_	1612.2	114.3	νCC_p_; δOH_p_
1593	1588.5	173.04	δOH_bp_; νCC_bp_; δCH_bp_; νN=C	1589.0	141.4	δOH_bp_; νCC_bp_; δCH_bp_; νN=C
1521/*1500*	1514.8	63.04	νCC_p_; δCH_p_; δOH_p_	1507.4	87.0	νCC_p_; δCH_p_; δOH_p_
1495	1496.9	81.64	δCH_bp_; δOH_bp_; νCC_bp_	1494.6	156.1	δCH_bp_; δOH_bp_; νCC_bp_
1477	1478.2	87.27	δCH_p-bp_; νCC_p-bp_; δOH_p-bp_	1485.5	10.7	δCH_p-bp_; νCC_p-bp_; δOH_p-bp_
1461	1464.1	52.23	νCC_bp_; δCH_bp_; δOH_bp_	1469.6	15.5	νCC_bp_; δCH_bp_; δOH_bp_
1421	1418.2	25.92	νCC_bp_; δCH_bp_; δOH_bp_; δCH_am_	1419.1	31.2	νCC_bp_; δCH_bp_; δOH_bp_; δCH_am_
*1368*/1358	1371.9	40.64	δCH_am_	1373.8	34.5	δCH_am_
1339	1338.8	2.96	δCH_p_; νCC_p_	1338.8	16.5	δCH_p_; νCC_p_
1331/1326	1334.8	2.83	νCC_bp_; δCH_bp_	1334.9	5.0	νCC_bp_; δCH_bp_
	1326.2	1.70	δCC_p_; δCH_p_; δOH_p_	1318.7	92.2	δCC_p_; δCH_p_; δOH_p_
1314	1302.9	131.61	νCO_bp_; δCC_bp_; δCH_bp_	1304.2	79.5	νCO_bp_; δCC_bp_; δCH_bp_
1308/*1285*	1292.9	28.68	νCO_p_; νC-N; δCC_p_; δCH_p_	1282.7	47.5	νCO_p_; νC-N; δCC_p_; δCH_p_
1264	1249.7	9.74	δCH_bp_	1250.8	17.9	δCH_bp_
1224/1217	1215.4	56.05	νCC_am_; δCH_p-bp_	1215.4	57.5	νCC_am_; δCH_p-bp_
*1196*/1181	1186.9	169.23	δOH_p_; δCH_p_	1194.6	41.4	δOH_p_; δCH_p_
1166	1178.0	28.77	δCH_p_; δOH_p_	1175.7	36.2	δCH_p_; δOH_p_
1158	1158.5	160.77	δCH_p_; νC_p_-N; νCO_p_	1156.5	192.3	δCH_p_; νC_p_-N; νCO_p_
1147	1144.6	7.08	δCH_bp_	1144.6	8.9	δCH_bp_
1093	1095.7	13.00	δCH_p_	1097.4	10.8	δCH_p_
1073	1084.1	15.73	νC-Br; δCC_bp_; δCH_bp_	1085.2	15.9	νC-Br; δCC_bp_; δCH_bp_
998	1005.4	7.15	δCC_p_; γCH_am_	1007.9	2.2	δCC_p_; γCH_am_
	994.4	10.83	γCH_am_	998.2	9.1	γCH_am_
973	973.5	0.10	γCH_p_	970.2	1.7	γCH_p_
957	969.8	53.61	δCC_p_; δCH_p_; νCO_p_	978.2	94.9	δCC_p_; δCH_p_; νCO_p_
	958.6	0.04	γCH_bp_	958.4	0.5	γCH_bp_
897	919.5	17.42	δCC_bp_; δCNC; δCCN_am_; νCBr	917.1	16.2	δCC_bp_; δCNC; δCCN_am_; νCBr
875	883.7	9.64	γCH_p-bp_	884.3	17.9	γCH_p-bp_
	880.0	5.20	γCH_p-bp_	874.1	8.2	γCH_p-bp_
859	856.2	42.42	γCC_p_; γCH_p-bp_; γOH_bp_	866.3	12.8	γCC_p_; γCH_p_
848	848.8	4.55	γOH_bp_	851.8	16.9	γCH_bp_; γOH_bp_
844	827.8	83.95	γCC_bp_; γCH_bp_; γOH_bp_	828.0	80.9	γCC_bp_; γCH_bp_; γOH_bp_
824/*805*	813.2	7.16	δCC_bp_; δCNC; δCCN_am_	805.0	7.9	δCC_bp_; δCNC; δCCN_am_
765	780.4	20.54	γCH_p_	779.0	11.8	γCH_p_; δCC_bp_
758	774.9	6.42	γCH_p_; δCC_bp_	775.9	34.0	γCH_p_
717	729.0	1.13	γCH_bp_; γCC_bp_	728.8	1.5	γCH_bp_; γCC_bp_
685	695.7	25.80	γCC_p_; δCC_bp_	694.3	22.9	γCC_p_; δCC_bp_
	686.0	16.75	γCC_p_	685.2	15.2	γCC_p_
633	635.8	16.53	γCC_p_; δCC_bp_; γCH_p_; νCBr	635.4	22.2	γCC_p_; δCC_bp_; γCH_p_; νCBr
610	624.7	8.06	γCC_p_; δCC_bp_; γCH_p_; νCBr	623.8	5.7	γCC_p_; δCC_bp_; γCH_p_; νCBr
571/*560*	574.1	10.14	δCC_p-bp_; δCNC; δCCN_am_	563.8	9.9	δCC_p-bp_; δCNC; δCCN_am_
553	552.7	4.79	γCC_bp_; γCH_bp_; γCH_am_	555.2	6.1	γCC_bp_; γCH_bp_; γCH_am_
541	530.8	1.48	δCC_p_	533.7	1.3	δCC_p_
*528*/521	516.2	17.41	δCN_p_	520.8	2.6	δCN_p_

*^a^* Frequencies (ν, in cm^−1^) were scaled by 0.994 or 0.945, below and above 1800 cm^−1^, respectively. Infrared intensities (I^IR^) in km mol^−1^. For full calculated spectra of the conformers see Table S2, in the Supporting Information. *^b^* Values in *italic* correspond to absorptions tentatively assigned to conformer II; values in round style are assigned to both conformers or to conformer V only (when a corresponding band ascribed to conformer II is indicated). *^c^* Approximate descriptions were obtained by visual inspection of the animated vibrations using GaussView 5.0. ν, stretching; δ, bending; γ, rocking/torsion; n.o., not observed. Subscript “p” refers to the phenol ring, “bp” to the bromo-substituted phenol ring, “am” to the azomethine bridging group.

## Data Availability

The data presented in this study are available in Supporting Information.

## Supplementary Materials

The following are available online. Figures S1–S3, with the IR spectrum of the compound (in KBr pellet; room temperature) and 1H and 13C NMR spectra (in DMSO; room temperature); Figure S4, with calculated IR spectra of the isomeric forms of the enol-imine and keto-amine tautomers of BHAP vs. the experimental matrix isolation IR spectrum of the compound; Table S1, with the Cartesian coordinates of the optimized calculated conformers of the *E*- and *Z*- isomers of the enol-imine and keto-amine tautomers of BHAP; Table S2, with the calculated IR spectra of the 4 most stable conformers of the experimentally relevant *E*-enol-imine isomer of BHAP.
