# Supplementary material for: Structural Relevance of Intramolecular H-Bonding in Ortho-Hydroxyaryl Schiff Bases: The Case of 3-(5-bromo-2-hydroxybenzylideneamino) Phenol
Source: Molecules. 2021 May 10;26(9):2814. doi: 10.3390/molecules26092814 (PMC8126046; doi:10.3390/molecules26092814)
Supplement: Supplementary file 1 [file molecules-26-02814-s001.zip › molecules-1210174-supplementary.pdf]

Supporting Information for

Structural relevance of intramolecular H-bonding in *ortho*-hydroxyaryl Schiff bases: the case of 3-(5-bromo-2-hydroxybenzylideneamino)phenol

İsa Sıdır,[1,2,*] Yadigar Gülseven Sıdır,[1,2] Sándor Góbi,[2,3] Halil Berber,[4] Rui Fausto[2,*]

[1] *Department of Physics, Faculty of Science and Arts, Bitlis Eren University, 13000 Bitlis, Turkey.*
[2] *CQC, Department of Chemistry, University of Coimbra, 3004-535 Coimbra, Portugal.*
[3] *MTA-ELTE Lendület Laboratory Astrochemistry Research Group, Institute of Chemistry.*
ELTE Eötvös Loránd University, H-1518 Budapest, Hungary.
[4] *Eskişehir Technical University, Faculty of Science, Department of Chemistry, 26470, Eskişehir, Turkey.*

INDEX

Figure S1. IR spectrum of BHAP in a KBr pellet at room temperature 2

Figure S2. ^{1}H-NMR spectrum of BHAP in DMSO-d_6, at room temperature 2

Figure S3. ^{13}C-NMR spectrum of BHAP in DMSO-d_6, at room temperature 3

Figure S4. Comparison between the matrix isolation IR spectrum of BHAP and the simulated spectra based on the DFT(B3LYP)/6-311++G(d,p) calculated IR spectra of the relevant conformers of *E*- and *Z*- geometrical isomers of the enol-imine and keto-amine tautomers of the compound 4

Table S1. Cartesian coordinates of the optimized structures of the conformers of BHAP isomers 5

Table S2. Calculated IR spectra of conformers I, II, V and VI (BHAP *E*-enol-imine isomer) 17

* Corresponding authors: İsa Sıdır: isidir@beu.edu.tr; Rui Fausto: rfausto@ci.uc.pt

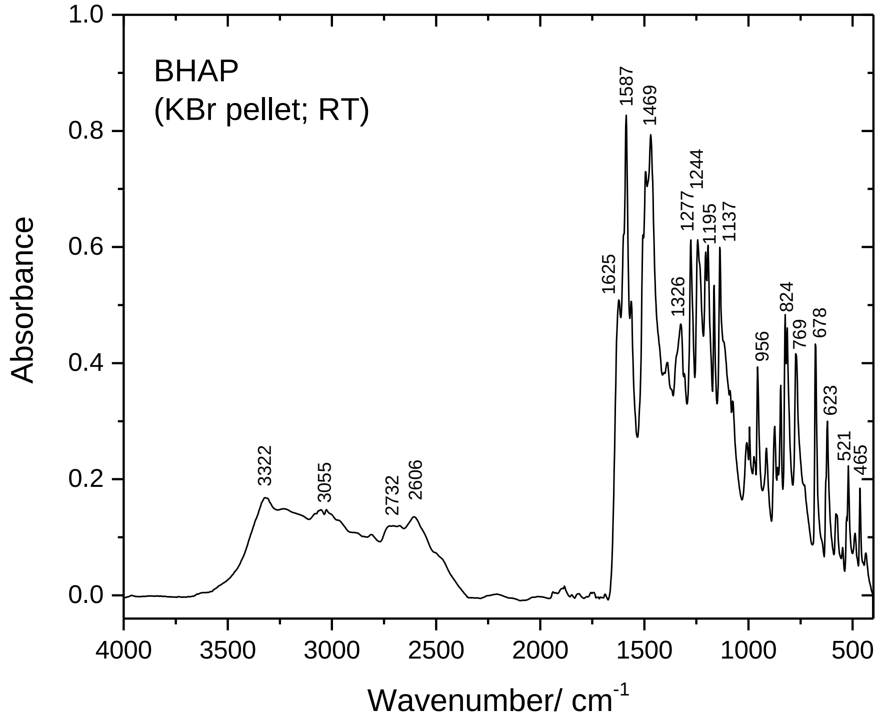

Figure S1. IR spectrum of BHAP in a KBr pellet at room temperature (RT).

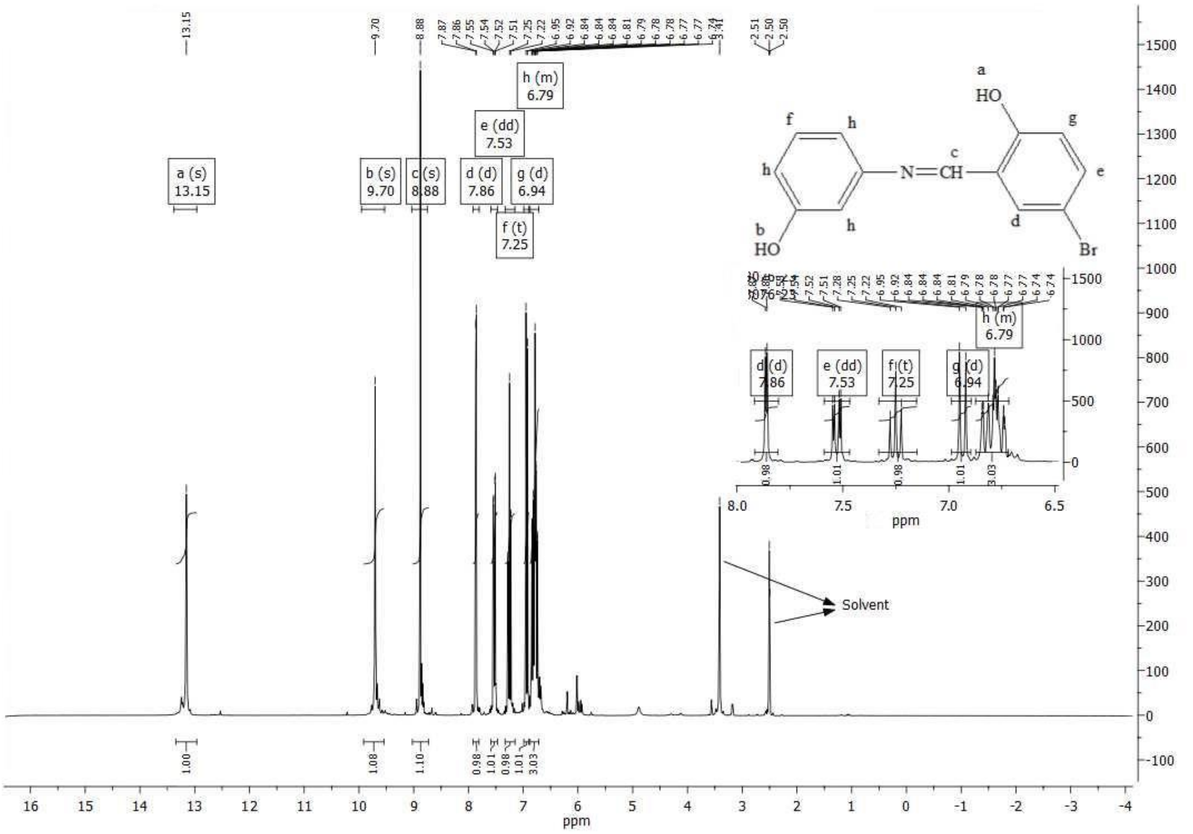

Figure S2. ^{1}H-NMR spectrum of BHAP in DMSO-d$_6$, at room temperature.

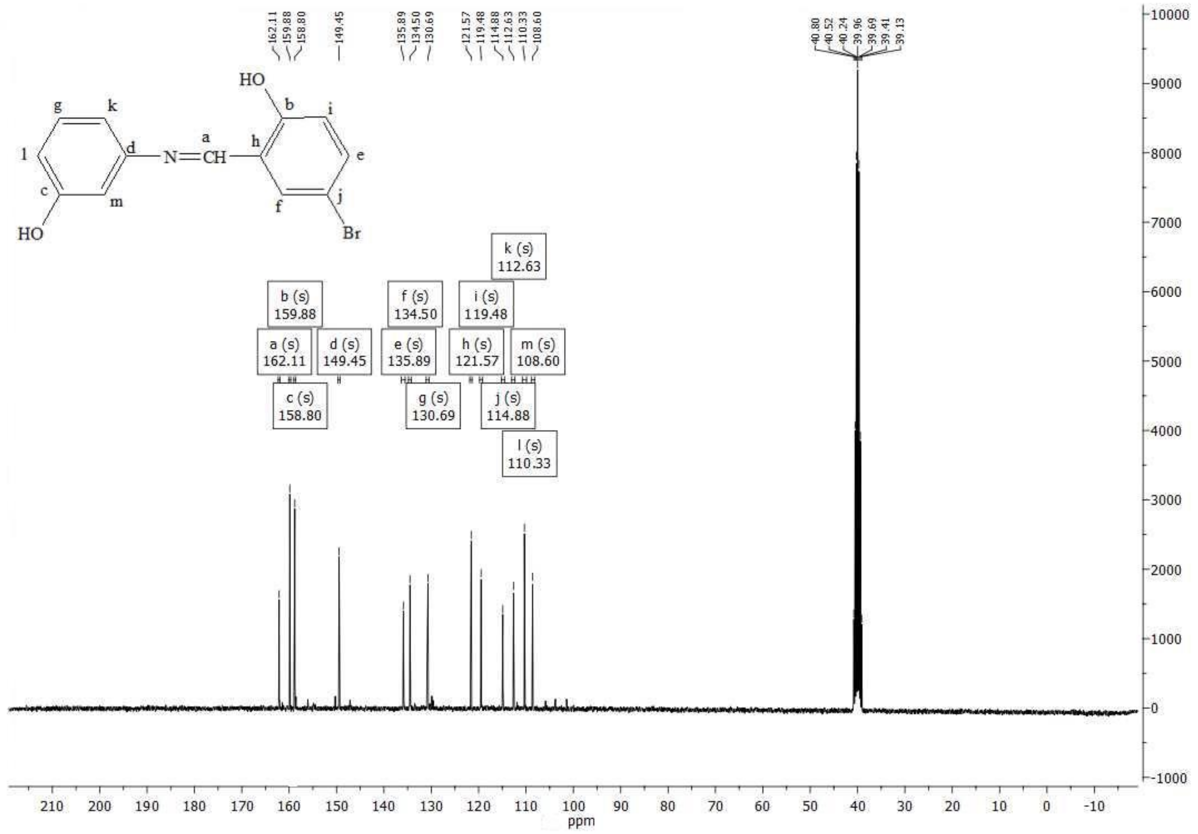

Figure S3. ^{13}C-NMR spectrum of BHAP in DMSO-d_6, at room temperature.

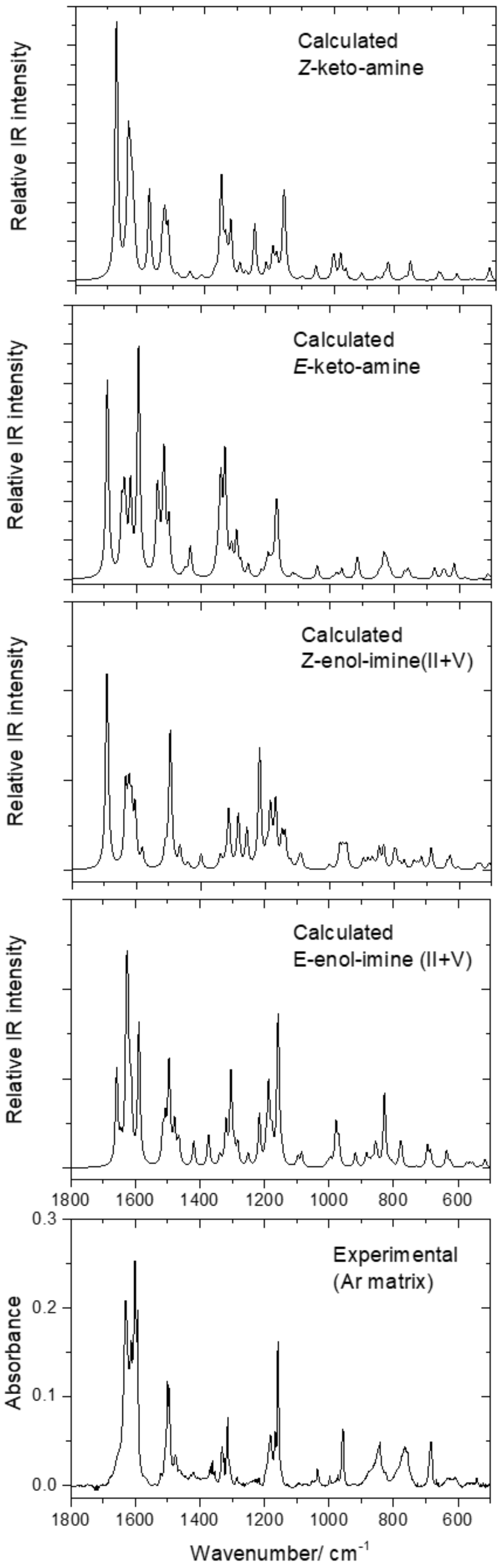

Figure S4. Comparison between the matrix isolation IR spectrum of BHAP (*bottom panel*) and the simulated spectra based on the DFT(B3LYP)/6-311++G(d,p) calculated IR spectra of the relevant conformers of *E*- and *Z*- geometrical isomers of the enol-imine and keto-amine tautomers of the compound. For *E*-keto-amine and *Z*-keto-amine forms, the pairs of conformers IV and VII, and II and V of the "*keto-amine forms of the E-enol-imine isomer*" (see Figure 3 and Table 4 of the article) were considered.

Table S1. Cartesian coordinates (Å) of the optimized structures of the conformers of BHAP isomers.

E-enol-imine I

6	5.279746000	0.739249000	0.842961000
6	5.666919000	-0.498200000	0.338904000
6	4.717654000	-1.303459000	-0.292985000
6	3.394030000	-0.880281000	-0.405041000
6	3.009772000	0.363370000	0.118780000
6	3.965747000	1.184273000	0.726798000
7	1.689064000	0.843963000	0.019939000
6	0.679027000	0.055511000	0.140218000
6	-0.687401000	0.519103000	-0.015572000
8	5.148198000	-2.500047000	-0.797862000
6	-1.747296000	-0.393303000	0.142448000
6	-3.055677000	0.023302000	-0.006762000
6	-3.345366000	1.356526000	-0.314476000
6	-2.314369000	2.270141000	-0.471152000
6	-0.979195000	1.872999000	-0.325727000
8	-0.015385000	2.789569000	-0.484167000
35	-4.488406000	-1.233822000	0.207566000
1	6.016948000	1.369792000	1.326672000
1	6.690217000	-0.845037000	0.408973000
1	2.671286000	-1.494901000	-0.932533000
1	3.663722000	2.149799000	1.112569000
1	0.811286000	-1.005282000	0.382152000
1	4.417004000	-2.962134000	-1.220854000
1	-1.524660000	-1.426420000	0.381992000
1	-4.373485000	1.675563000	-0.429698000
1	-2.522109000	3.306330000	-0.708290000
1	0.856508000	2.336754000	-0.339486000

E-enol-imine II

6	5.278080000	0.754509000	0.820823000
6	5.661841000	-0.491736000	0.326690000
6	4.709419000	-1.305203000	-0.289575000
6	3.385483000	-0.884134000	-0.400454000
6	3.005907000	0.361357000	0.112042000
6	3.964722000	1.193009000	0.708209000
7	1.685034000	0.843483000	0.016362000
6	0.676170000	0.052744000	0.133089000
6	-0.690572000	0.517199000	-0.016873000
8	5.015501000	-2.529801000	-0.818245000
6	-1.749811000	-0.396954000	0.135360000
6	-3.058591000	0.021005000	-0.006815000
6	-3.349248000	1.356724000	-0.302319000
6	-2.318887000	2.271981000	-0.453307000
6	-0.983350000	1.873550000	-0.315016000
8	-0.019694000	2.791415000	-0.467786000
35	-4.491091000	-1.237876000	0.199911000
1	6.018436000	1.390123000	1.292793000
1	6.693423000	-0.820547000	0.405272000
1	2.678459000	-1.519965000	-0.918554000
1	3.662742000	2.161982000	1.085109000
1	0.810249000	-1.009328000	0.366507000
1	5.954948000	-2.708557000	-0.705855000
1	-1.526072000	-1.432211000	0.364119000
1	-4.377731000	1.676538000	-0.412181000
1	-2.527324000	3.310050000	-0.681310000
1	0.852376000	2.335964000	-0.330194000

E-enol-imine III

6	5.250087000	0.545654000	1.067422000
6	5.617738000	-0.622553000	0.404438000
6	4.677828000	-1.273080000	-0.395043000
6	3.381942000	-0.772198000	-0.515097000
6	3.015819000	0.400822000	0.164227000
6	3.969065000	1.073139000	0.940421000
7	1.734232000	0.964925000	0.055506000
6	0.705551000	0.212936000	0.100833000
6	-0.683620000	0.659557000	-0.051803000
8	5.086489000	-2.404880000	-1.051352000
6	-1.680877000	-0.315790000	0.120057000
6	-3.025598000	-0.006311000	-0.001918000
6	-3.422894000	1.292312000	-0.303403000
6	-2.451999000	2.271301000	-0.479619000
6	-1.090375000	1.978470000	-0.360273000
8	-0.149470000	2.936597000	-0.538948000
35	-4.346764000	-1.373652000	0.244600000
1	5.981217000	1.058479000	1.682290000
1	6.619145000	-1.027567000	0.479326000
1	2.666349000	-1.265679000	-1.165744000
1	3.683589000	1.991002000	1.438336000
1	0.802501000	-0.866804000	0.287722000
1	4.361321000	-2.756224000	-1.578158000
1	-1.380438000	-1.329648000	0.355512000
1	-4.471512000	1.541505000	-0.400485000
1	-2.757027000	3.286400000	-0.716711000
1	-0.580182000	3.772588000	-0.750697000

E-enol-imine IV

6	5.247716000	0.557065000	1.056449000
6	5.611422000	-0.616739000	0.394466000
6	4.668439000	-1.271831000	-0.398054000
6	3.372582000	-0.771966000	-0.516805000
6	3.011562000	0.400139000	0.159487000
6	3.967337000	1.079099000	0.932287000
7	1.730398000	0.966833000	0.053836000
6	0.702572000	0.213605000	0.098930000
6	-0.687145000	0.659790000	-0.050679000
8	4.958664000	-2.415848000	-1.095423000
6	-1.682947000	-0.317738000	0.117187000
6	-3.028098000	-0.009230000	-0.002699000
6	-3.427225000	1.289896000	-0.299366000
6	-2.457670000	2.270885000	-0.472003000
6	-1.095702000	1.979317000	-0.353680000
8	-0.156402000	2.940148000	-0.527909000
35	-4.347841000	-1.378830000	0.239209000
1	5.982024000	1.072079000	1.665605000
1	6.620868000	-1.007345000	0.480008000
1	2.671655000	-1.282697000	-1.165429000
1	3.681641000	1.998430000	1.427339000
1	0.801283000	-0.866338000	0.281311000
1	5.879063000	-2.658145000	-0.950289000
1	-1.380765000	-1.332399000	0.346800000
1	-4.476206000	1.537951000	-0.395582000
1	-2.764024000	3.286458000	-0.705482000
1	-0.589035000	3.775059000	-0.739943000

E-enol-imine V

6	−5.272699000	−0.021221000	0.442529000
6	−5.515645000	−1.263353000	−0.149386000
6	−4.466774000	−1.938977000	−0.762208000
6	−3.178969000	−1.406450000	−0.783353000
6	−2.936943000	−0.167681000	−0.175279000
6	−3.995174000	0.532116000	0.418774000
7	−1.669682000	0.448870000	−0.169721000
6	−0.593077000	−0.244373000	−0.037142000
6	0.725571000	0.359996000	−0.088983000
6	1.863089000	−0.451853000	0.078243000
6	3.128248000	0.100459000	0.030229000
6	3.296390000	1.472284000	−0.183899000
6	2.187413000	2.287962000	−0.348644000
6	0.894061000	1.752914000	−0.303601000
8	−0.149183000	2.578161000	−0.464404000
35	4.669421000	−1.018478000	0.258070000
8	−6.334143000	0.612416000	1.026913000
1	−6.519124000	−1.669609000	−0.132185000
1	−4.657733000	−2.892203000	−1.242103000
1	−2.381573000	−1.926019000	−1.300056000
1	−3.798024000	1.499563000	0.869490000
1	−0.631470000	−1.325630000	0.134939000
1	1.734408000	−1.515060000	0.243853000
1	4.291363000	1.897530000	−0.220501000
1	2.300955000	3.352262000	−0.514350000
1	−0.975498000	2.030696000	−0.399332000
1	−6.051604000	1.457546000	1.392073000

E-enol-imine VI

6	−5.264691000	−0.018394000	−0.453616000
6	−5.507853000	−1.250841000	0.158285000
6	−4.460259000	−1.917851000	0.789481000
6	−3.177096000	−1.382106000	0.805546000
6	−2.936411000	−0.150340000	0.175800000
6	−3.988479000	0.538131000	−0.434069000
7	−1.667342000	0.463118000	0.169192000
6	−0.593615000	−0.233642000	0.036391000
6	0.727258000	0.366221000	0.086558000
6	1.861706000	−0.450066000	−0.079315000
6	3.128846000	0.097572000	−0.031809000
6	3.302058000	1.469118000	0.179971000
6	2.196117000	2.289100000	0.342965000
6	0.900501000	1.758893000	0.298979000
8	−0.138656000	2.588555000	0.458864000
35	4.666018000	−1.027767000	−0.257310000
8	−6.245084000	0.696380000	−1.084697000
1	−6.506518000	−1.676382000	0.153892000
1	−4.653351000	−2.862779000	1.284573000
1	−2.379819000	−1.890556000	1.333317000
1	−3.812326000	1.496977000	−0.904873000
1	−0.635529000	−1.315033000	−0.135113000
1	1.729038000	−1.513012000	−0.243447000
1	4.298547000	1.890878000	0.215731000
1	2.313439000	3.353339000	0.506342000
1	−0.968397000	2.046738000	0.391896000
1	−7.082894000	0.225622000	−1.025161000

E-enol-imine VII

6	5.257719000	−0.145636000	0.502629000
6	5.459419000	−1.321092000	−0.225428000
6	4.401428000	−1.855839000	−0.951937000
6	3.145566000	−1.251173000	−0.950311000
6	2.943152000	−0.078652000	−0.207167000
6	4.016480000	0.483366000	0.498465000
7	1.720104000	0.612653000	−0.176521000
6	0.630308000	−0.044466000	−0.090378000
6	−0.717999000	0.533469000	−0.109182000
6	−1.787183000	−0.364482000	0.051898000
6	−3.103479000	0.066855000	0.046087000
6	−3.399717000	1.415569000	−0.122050000
6	−2.356597000	2.320241000	−0.283495000
6	−1.022236000	1.903817000	−0.280990000
8	−0.011488000	2.792170000	−0.442430000
35	−4.523466000	−1.201542000	0.269971000
8	6.327936000	0.353640000	1.196822000
1	6.438107000	−1.784341000	−0.222397000
1	4.560145000	−2.755295000	−1.536521000
1	2.340857000	−1.658961000	−1.549698000
1	3.853130000	1.407427000	1.043686000
1	0.637955000	−1.137861000	0.024159000
1	−1.565390000	−1.416603000	0.183036000
1	−4.425591000	1.760313000	−0.127720000
1	−2.582620000	3.374331000	−0.415893000
1	−0.379280000	3.674649000	−0.565783000
1	6.070518000	1.163410000	1.649805000

E-enol-imine VIII

6	5.244724000	−0.144949000	0.521449000
6	5.452628000	−1.301209000	−0.235863000
6	4.402074000	−1.818819000	−0.990444000
6	3.150197000	−1.212684000	−0.983865000
6	2.943260000	−0.057287000	−0.210305000
6	4.004772000	0.486264000	0.521188000
7	1.717006000	0.628422000	−0.180249000
6	0.630862000	−0.033555000	−0.093243000
6	−0.720322000	0.538471000	−0.109408000
6	−1.785586000	−0.364223000	0.049405000
6	−3.103473000	0.062330000	0.047947000
6	−3.404948000	1.410790000	−0.112587000
6	−2.365587000	2.320140000	−0.271808000
6	−1.029589000	1.908532000	−0.274524000
8	−0.022164000	2.799963000	−0.434509000
35	−4.518591000	−1.212491000	0.268113000
8	6.235109000	0.423301000	1.278897000
1	6.426540000	−1.781005000	−0.246690000
1	4.567767000	−2.702848000	−1.596142000
1	2.349577000	−1.602973000	−1.600096000
1	3.857058000	1.392512000	1.094324000
1	0.643547000	−1.127119000	0.021378000
1	−1.559580000	−1.416031000	0.175843000
1	−4.432091000	1.751795000	−0.114200000
1	−2.595683000	3.374026000	−0.398509000
1	−0.391127000	3.684019000	−0.541955000
1	7.047996000	−0.083068000	1.181077000

E-enol-imine IX

6	-4.672591000	-1.681090000	1.075823000
6	-5.469566000	-0.941497000	0.206405000
6	-4.867773000	-0.017916000	-0.648968000
6	-3.487213000	0.177095000	-0.620178000
6	-2.691639000	-0.565424000	0.267592000
6	-3.291030000	-1.517012000	1.101950000
7	-1.294829000	-0.423257000	0.320769000
6	-0.761007000	0.734741000	0.261027000
6	0.688567000	0.942962000	0.217596000
8	-5.685466000	0.667081000	-1.507482000
6	1.254628000	2.228716000	0.329544000
6	2.638384000	2.397662000	0.234639000
6	3.469836000	1.305330000	0.044866000
6	2.912672000	0.026639000	-0.047660000
6	1.547378000	-0.158031000	0.035501000
8	0.528935000	3.368816000	0.528939000
35	4.066327000	-1.484015000	-0.304368000
1	-5.137457000	-2.406552000	1.733768000
1	-6.542600000	-1.079839000	0.163027000
1	-3.021243000	0.868692000	-1.315578000
1	-2.665381000	-2.101438000	1.764468000
1	-1.401197000	1.629590000	0.230432000
1	-5.161300000	1.249258000	-2.067250000
1	3.045069000	3.397622000	0.321501000
1	4.541120000	1.442674000	-0.025718000
1	1.103818000	-1.141482000	-0.044794000
1	-0.390082000	3.164575000	0.727146000

E-enol-imine X

6	-4.597741000	-1.890651000	0.871912000
6	-5.454771000	-1.020696000	0.196991000
6	-4.915748000	0.056313000	-0.508201000
6	-3.539364000	0.272679000	-0.527670000
6	-2.685638000	-0.597171000	0.162698000
6	-3.222696000	-1.698962000	0.846964000
7	-1.288039000	-0.446029000	0.162526000
6	-0.769458000	0.719255000	0.237172000
6	0.683879000	0.923071000	0.182978000
8	-5.693010000	0.938442000	-1.212898000
6	1.227915000	2.215243000	0.300931000
6	2.609854000	2.405089000	0.249369000
6	3.464519000	1.322058000	0.080520000
6	2.925717000	0.041349000	-0.036922000
6	1.557666000	-0.161578000	0.011694000
8	0.362557000	3.260440000	0.465370000
35	4.104355000	-1.454416000	-0.271263000
1	-5.014001000	-2.733226000	1.412438000
1	-6.527498000	-1.188024000	0.204731000
1	-3.147643000	1.098132000	-1.109002000
1	-2.549843000	-2.377982000	1.355105000
1	-1.377867000	1.619177000	0.361084000
1	-6.618986000	0.685878000	-1.137424000
1	3.021978000	3.405483000	0.341100000
1	4.535200000	1.474811000	0.041347000
1	1.128622000	-1.150723000	-0.078724000
1	0.852704000	4.086186000	0.535481000

E-enol-imine XI

6	-4.636524000	-1.766320000	1.001811000
6	-5.457257000	-0.970031000	0.202201000
6	-4.880554000	0.016863000	-0.598190000
6	-3.501118000	0.217748000	-0.589367000
6	-2.685257000	-0.576253000	0.226685000
6	-3.259220000	-1.590400000	1.008973000
7	-1.287796000	-0.431936000	0.262859000
6	-0.758982000	0.729840000	0.246153000
6	0.689362000	0.944527000	0.201774000
8	-5.618377000	0.820499000	-1.426092000
6	1.252012000	2.231469000	0.319796000
6	2.637180000	2.401635000	0.242830000
6	3.472239000	1.311323000	0.059978000
6	2.917560000	0.032366000	-0.045144000
6	1.552086000	-0.154241000	0.022752000
8	0.525680000	3.371492000	0.510496000
35	4.076515000	-1.475948000	-0.294211000
1	-5.083250000	-2.539275000	1.616732000
1	-6.531338000	-1.127448000	0.187957000
1	-3.077467000	0.962166000	-1.252312000
1	-2.614136000	-2.213059000	1.615599000
1	-1.404333000	1.620983000	0.262295000
1	-6.549639000	0.583832000	-1.363976000
1	3.041174000	3.402239000	0.334601000
1	4.544115000	1.450228000	0.002640000
1	1.110477000	-1.137984000	-0.063914000
1	-0.406653000	3.172980000	0.641011000

E-enol-imine XII

6	-4.602177000	-1.899580000	0.864030000
6	-5.461441000	-1.029328000	0.198972000
6	-4.924136000	0.051513000	-0.501384000
6	-3.547426000	0.268491000	-0.521601000
6	-2.687417000	-0.606486000	0.161618000
6	-3.223883000	-1.710015000	0.837191000
7	-1.290583000	-0.451303000	0.159276000
6	-0.772470000	0.713706000	0.241092000
6	0.680281000	0.920083000	0.184947000
8	-5.801669000	0.869122000	-1.164375000
6	1.222537000	2.212625000	0.306053000
6	2.604074000	2.404675000	0.256578000
6	3.460501000	1.323197000	0.086339000
6	2.923607000	0.042086000	-0.035782000
6	1.555776000	-0.162911000	0.011124000
8	0.355348000	3.257446000	0.469829000
35	4.104345000	-1.451081000	-0.273468000
1	-5.015562000	-2.745962000	1.400904000
1	-6.533611000	-1.180738000	0.196071000
1	-3.136412000	1.093775000	-1.094945000
1	-2.550621000	-2.393006000	1.339493000
1	-1.380870000	1.612494000	0.374760000
1	-5.317645000	1.568129000	-1.616048000
1	3.014746000	3.405414000	0.351147000
1	4.531001000	1.477544000	0.049024000
1	1.128335000	-1.152527000	-0.081659000
1	0.846248000	4.080935000	0.558993000

E-enol-imine XIII

6	−4.860181000	−1.057304000	0.398092000
6	−5.524352000	−0.163989000	−0.446987000
6	−4.783030000	0.765974000	−1.166585000
6	−3.396054000	0.833736000	−1.044596000
6	−2.734502000	−0.054497000	−0.183879000
6	−3.474111000	−1.016758000	0.517576000
7	−1.338727000	−0.072232000	−0.021848000
6	−0.682993000	1.021730000	0.038521000
6	0.779048000	1.067642000	0.119944000
6	1.472407000	2.275018000	0.338947000
6	2.869506000	2.284773000	0.377762000
6	3.587855000	1.111322000	0.213503000
6	2.902111000	−0.089787000	0.010072000
6	1.523390000	−0.117300000	−0.037997000
8	−5.627324000	−1.962501000	1.079238000
8	0.866327000	3.484318000	0.523345000
35	3.897145000	−1.714812000	−0.211756000
1	−6.601522000	−0.224642000	−0.538570000
1	−5.293284000	1.442642000	−1.842915000
1	−2.827943000	1.533960000	−1.644755000
1	−2.945675000	−1.715734000	1.157766000
1	−1.221640000	1.981007000	0.024473000
1	3.375687000	3.227387000	0.545529000
1	4.669625000	1.126400000	0.245850000
1	0.981505000	−1.038741000	−0.203216000
1	−5.063160000	−2.535656000	1.608975000
1	−0.089168000	3.389514000	0.584365000

E-enol-imine XIV

6	−4.856710000	−1.048371000	0.414001000
6	−5.515489000	−0.169422000	−0.450635000
6	−4.770351000	0.745174000	−1.191241000
6	−3.386291000	0.807973000	−1.066460000
6	−2.730751000	−0.068884000	−0.185044000
6	−3.470648000	−1.012887000	0.534322000
7	−1.334273000	−0.083647000	−0.026444000
6	−0.682432000	1.011640000	0.039436000
6	0.779820000	1.062221000	0.120619000
6	1.469560000	2.271994000	0.336393000
6	2.866619000	2.286273000	0.374010000
6	3.588288000	1.114601000	0.211744000
6	2.906059000	−0.089124000	0.011889000
6	1.527345000	−0.121068000	−0.034547000
8	−5.523884000	−1.978234000	1.164135000
8	0.859631000	3.480235000	0.517755000
35	3.906178000	−1.711396000	−0.206344000
1	−6.595256000	−0.211333000	−0.555242000
1	−5.278096000	1.409644000	−1.881335000
1	−2.812953000	1.494313000	−1.677507000
1	−2.966311000	−1.708474000	1.192693000
1	−1.224431000	1.969331000	0.031597000
1	3.370061000	3.230786000	0.539345000
1	4.670043000	1.132840000	0.243330000
1	0.987770000	−1.044684000	−0.195441000
1	−6.468138000	−1.926117000	0.983458000
1	−0.095083000	3.381352000	0.584230000

E-enol-imine XV

6	−4.836086000	−1.130199000	0.332739000
6	−5.544248000	−0.132049000	−0.340793000
6	−4.842114000	0.905530000	−0.944534000
6	−3.452272000	0.974263000	−0.873652000
6	−2.744929000	−0.021822000	−0.184186000
6	−3.446387000	−1.087583000	0.396680000
7	−1.342670000	−0.048530000	−0.085867000
6	−0.690674000	1.039739000	0.068807000
6	0.776968000	1.060208000	0.112999000
6	1.468849000	2.269825000	0.306993000
6	2.864093000	2.284200000	0.348580000
6	3.586553000	1.106010000	0.200093000
6	2.901145000	−0.093051000	0.007937000
6	1.518475000	−0.121588000	−0.037500000
8	−5.564341000	−2.139211000	0.904616000
35	3.895083000	−1.721357000	−0.196728000
8	0.731610000	3.412172000	0.449666000
1	−6.624064000	−0.193134000	−0.393319000
1	−5.387600000	1.669533000	−1.487302000
1	−2.917313000	1.769816000	−1.377421000
1	−2.884176000	−1.866772000	0.901320000
1	−1.190101000	2.004467000	0.190438000
1	3.390901000	3.221879000	0.497988000
1	4.668156000	1.123127000	0.233211000
1	0.976213000	−1.045500000	−0.187891000
1	−4.970942000	−2.767374000	1.329514000
1	1.315319000	4.166586000	0.580760000

E-enol-imine XVI

6	−4.832365000	−1.122420000	0.349299000
6	−5.534889000	−0.135427000	−0.347159000
6	−4.828842000	0.888997000	−0.974832000
6	−3.441671000	0.950913000	−0.901681000
6	−2.740590000	−0.036969000	−0.188536000
6	−3.442696000	−1.086040000	0.414074000
7	−1.337944000	−0.060410000	−0.092976000
6	−0.690227000	1.028560000	0.070196000
6	0.777676000	1.054038000	0.114646000
6	1.465573000	2.265640000	0.309123000
6	2.860718000	2.284449000	0.351141000
6	3.586815000	1.108519000	0.202421000
6	2.905237000	−0.092656000	0.009804000
6	1.522600000	−0.125613000	−0.035420000
8	−5.460179000	−2.162678000	0.981667000
35	3.904613000	−1.717515000	−0.194841000
8	0.724875000	3.406147000	0.451363000
1	−6.618041000	−0.175829000	−0.410389000
1	−5.371551000	1.642763000	−1.534348000
1	−2.900643000	1.733051000	−1.419729000
1	−2.904598000	−1.864202000	0.940066000
1	−1.193298000	1.990555000	0.199905000
1	3.384542000	3.223757000	0.500996000
1	4.668359000	1.128888000	0.235970000
1	0.982839000	−1.051311000	−0.184412000
1	−6.411782000	−2.092836000	0.853703000
1	1.306712000	4.161381000	0.585822000

Z-enol-imine I

6	3.182195000	-0.019281000	-2.024965000
6	4.373642000	-0.457057000	-1.456676000
6	4.638238000	-0.158065000	-0.118753000
6	3.721836000	0.575277000	0.635660000
6	2.508568000	0.990409000	0.061267000
6	2.243511000	0.695490000	-1.284410000
7	1.652475000	1.795866000	0.831128000
6	0.414195000	1.558019000	1.025059000
6	-0.452226000	0.402199000	0.681795000
8	5.823415000	-0.595991000	0.400069000
6	-1.786729000	0.725900000	0.366519000
6	-2.713360000	-0.257168000	0.073104000
6	-2.352138000	-1.603395000	0.121437000
6	-1.052476000	-1.941810000	0.462048000
6	-0.093222000	-0.961910000	0.743902000
8	1.117875000	-1.429208000	1.135435000
35	-4.511839000	0.229494000	-0.380158000
1	2.979171000	-0.241289000	-3.066586000
1	5.103813000	-1.015963000	-2.028035000
1	3.939152000	0.852588000	1.662437000
1	1.324578000	1.041295000	-1.740002000
1	-0.129564000	2.357683000	1.534335000
1	5.897650000	-0.330203000	1.322819000
1	-2.081563000	1.768541000	0.351018000
1	-3.078164000	-2.376131000	-0.095815000
1	-0.749129000	-2.979836000	0.522442000
1	1.818566000	-0.770095000	1.025076000

Z-enol-imine II

6	3.168426000	0.035928000	-2.025697000
6	4.352957000	-0.429371000	-1.456817000
6	4.614266000	-0.161991000	-0.111687000
6	3.703711000	0.566875000	0.654034000
6	2.503054000	1.007044000	0.080839000
6	2.238795000	0.745428000	-1.274979000
7	1.645573000	1.803056000	0.858839000
6	0.410443000	1.556224000	1.057605000
6	-0.449853000	0.398886000	0.702417000
8	5.755850000	-0.575323000	0.514294000
6	-1.784115000	0.720709000	0.384138000
6	-2.703798000	-0.262042000	0.069138000
6	-2.335387000	-1.606988000	0.097585000
6	-1.036652000	-1.944054000	0.442089000
6	-0.084111000	-0.963911000	0.747150000
8	1.124756000	-1.433746000	1.140053000
35	-4.502227000	0.222276000	-0.387445000
1	2.969218000	-0.162896000	-3.072686000
1	5.068258000	-0.986427000	-2.053517000
1	3.937209000	0.806288000	1.684122000
1	1.325904000	1.112260000	-1.726150000
1	-0.136528000	2.347593000	1.576349000
1	6.311694000	-1.059611000	-0.105272000
1	-2.083746000	1.762096000	0.382708000
1	-3.055873000	-2.379898000	-0.136820000
1	-0.728828000	-2.981443000	0.489500000
1	1.820374000	-0.762901000	1.080808000

Z-enol-imine III

6	2.881219000	-0.133789000	-2.047450000
6	4.135778000	-0.537645000	-1.603433000
6	4.552469000	-0.162643000	-0.323842000
6	3.728202000	0.605077000	0.492577000
6	2.449635000	0.972312000	0.054267000
6	2.031016000	0.612415000	-1.233372000
7	1.683078000	1.802469000	0.890413000
6	0.461160000	1.598375000	1.181746000
6	-0.440304000	0.457307000	0.857691000
8	5.799340000	-0.570002000	0.075752000
6	-1.738879000	0.761398000	0.427718000
6	-2.634278000	-0.246911000	0.099648000
6	-2.269445000	-1.583780000	0.222599000
6	-0.999126000	-1.898534000	0.692832000
6	-0.087062000	-0.893740000	1.020897000
8	1.139332000	-1.174886000	1.537967000
35	-4.392184000	0.204950000	-0.518560000
1	2.560148000	-0.402099000	-3.047988000
1	4.802043000	-1.117288000	-2.230025000
1	4.054488000	0.918618000	1.479042000
1	1.063750000	0.934477000	-1.597325000
1	-0.027593000	2.397910000	1.745172000
1	5.984345000	-0.225291000	0.955583000
1	-2.033446000	1.799998000	0.333910000
1	-2.967484000	-2.371612000	-0.028633000
1	-0.717414000	-2.939801000	0.816935000
1	1.294376000	-2.125408000	1.529011000

Z-enol-imine IV

6	-2.896996000	0.326313000	2.030077000
6	-4.142541000	-0.181372000	1.665704000
6	-4.543832000	-0.104095000	0.329100000
6	-3.715995000	0.475607000	-0.626338000
6	-2.451769000	0.946219000	-0.260013000
6	-2.044977000	0.882840000	1.082236000
7	-1.677770000	1.593722000	-1.238467000
6	-0.454726000	1.338101000	-1.476717000
6	0.443462000	0.281666000	-0.931604000
8	-5.758010000	-0.576577000	-0.099853000
6	1.741202000	0.661655000	-0.564211000
6	2.634266000	-0.265531000	-0.045808000
6	2.267921000	-1.600836000	0.089423000
6	0.998126000	-1.998931000	-0.314415000
6	0.087922000	-1.074948000	-0.831554000
8	-1.137405000	-1.447146000	-1.288189000
35	4.391216000	0.295579000	0.478947000
1	-2.590073000	0.287629000	3.069434000
1	-4.800283000	-0.617003000	2.411741000
1	-4.048169000	0.553098000	-1.653527000
1	-1.085515000	1.288741000	1.376234000
1	0.038392000	2.012344000	-2.182288000
1	-6.261811000	-0.900721000	0.653664000
1	2.037778000	1.698302000	-0.672533000
1	2.964461000	-2.326310000	0.488988000
1	0.715546000	-3.044493000	-0.237464000
1	-1.287553000	-2.383245000	-1.119052000

Z-enol-imine V

6	-3.436112000	1.518118000	-0.299785000
6	-4.570276000	0.994747000	0.327255000
6	-4.739517000	-0.383059000	0.378287000
6	-3.803879000	-1.247602000	-0.190594000
6	-2.653434000	-0.719519000	-0.794688000
6	-2.477736000	0.672393000	-0.854782000
7	-1.752650000	-1.596196000	-1.423977000
6	-0.501983000	-1.644360000	-1.179355000
6	0.345418000	-0.964186000	-0.166731000
6	1.643093000	-0.627476000	-0.599798000
6	2.548830000	-0.020761000	0.250315000
6	2.205885000	0.237148000	1.577574000
6	0.945807000	-0.118754000	2.030281000
6	0.006276000	-0.717519000	1.181514000
8	-1.157037000	-1.083525000	1.771685000
35	4.292759000	0.451066000	-0.392549000
8	-3.315259000	2.878479000	-0.336315000
1	-5.298673000	1.673301000	0.752678000
1	-5.622395000	-0.793553000	0.854768000
1	-3.955570000	-2.319943000	-0.188905000
1	-1.601692000	1.081389000	-1.347181000
1	0.070323000	-2.295500000	-1.844913000
1	1.925462000	-0.846879000	-1.622716000
1	2.916382000	0.700787000	2.249704000
1	0.659377000	0.053603000	3.060554000
1	-1.870729000	-1.227082000	1.132364000
1	-2.496375000	3.125313000	-0.779188000

Z-enol-imine VI

6	-3.466753000	1.492512000	-0.337976000
6	-4.591076000	0.965522000	0.303732000
6	-4.740715000	-0.416096000	0.389023000
6	-3.793148000	-1.274688000	-0.160748000
6	-2.650323000	-0.738984000	-0.780319000
6	-2.495096000	0.650165000	-0.874182000
7	-1.740360000	-1.617689000	-1.391616000
6	-0.489127000	-1.651498000	-1.148131000
6	0.357247000	-0.951526000	-0.148756000
6	1.655942000	-0.627443000	-0.588451000
6	2.563622000	-0.006752000	0.249056000
6	2.221699000	0.279519000	1.570594000
6	0.960554000	-0.062925000	2.030331000
6	0.019427000	-0.675707000	1.193992000
8	-1.145585000	-1.025952000	1.792047000
35	4.309627000	0.444692000	-0.402747000
8	-3.259267000	2.836760000	-0.469404000
1	-5.342866000	1.627619000	0.721452000
1	-5.616640000	-0.827888000	0.876990000
1	-3.926143000	-2.349116000	-0.133565000
1	-1.635486000	1.080482000	-1.371747000
1	0.086627000	-2.308498000	-1.804928000
1	1.937122000	-0.867435000	-1.606999000
1	2.933561000	0.755103000	2.232826000
1	0.674520000	0.132138000	3.056649000
1	-1.857322000	-1.183289000	1.154462000
1	-3.982513000	3.321396000	-0.057830000

Z-enol-imine VII

6	-3.164422000	1.649358000	-0.134418000
6	-4.387765000	1.216293000	0.376479000
6	-4.729844000	-0.128988000	0.257825000
6	-3.878268000	-1.037557000	-0.361489000
6	-2.634915000	-0.605752000	-0.838831000
6	-2.286861000	0.748213000	-0.736043000
7	-1.819493000	-1.527738000	-1.517581000
6	-0.578912000	-1.698620000	-1.292494000
6	0.312800000	-1.108046000	-0.254395000
6	1.574426000	-0.657709000	-0.665389000
6	2.457020000	-0.087491000	0.241520000
6	2.117952000	0.017830000	1.586784000
6	0.887023000	-0.467748000	2.015085000
6	-0.012790000	-1.037270000	1.111781000
8	-1.197429000	-1.570462000	1.511528000
35	4.159477000	0.554299000	-0.363772000
8	-2.868855000	2.983551000	-0.017252000
1	-5.054808000	1.935031000	0.835867000
1	-5.686560000	-0.468327000	0.639298000
1	-4.151681000	-2.078849000	-0.474342000
1	-1.342382000	1.095265000	-1.142322000
1	-0.063384000	-2.371304000	-1.983315000
1	1.849972000	-0.742454000	-1.710050000
1	2.806467000	0.457368000	2.296687000
1	0.627854000	-0.413788000	3.068107000
1	-1.342220000	-1.401263000	2.448572000
1	-2.010912000	3.162233000	-0.416130000

Z-enol-imine VIII

6	-3.206286000	1.626971000	-0.159560000
6	-4.423116000	1.177071000	0.353114000
6	-4.741391000	-0.178878000	0.254493000
6	-3.869406000	-1.075390000	-0.346808000
6	-2.631543000	-0.622384000	-0.828299000
6	-2.307994000	0.735555000	-0.743995000
7	-1.803547000	-1.537271000	-1.500437000
6	-0.560784000	-1.692367000	-1.277142000
6	0.328597000	-1.091785000	-0.243981000
6	1.591432000	-0.648868000	-0.659789000
6	2.476481000	-0.073653000	0.240971000
6	2.138656000	0.045623000	1.585272000
6	0.906458000	-0.431574000	2.018572000
6	0.004401000	-1.006630000	1.121358000
8	-1.181331000	-1.533402000	1.529131000
35	4.181500000	0.554689000	-0.370652000
8	-2.831010000	2.945803000	-0.116089000
1	-5.120433000	1.876377000	0.804415000
1	-5.692749000	-0.530193000	0.638358000
1	-4.120664000	-2.123571000	-0.446301000
1	-1.377371000	1.114245000	-1.146833000
1	-0.039278000	-2.359913000	-1.968585000
1	1.865513000	-0.743306000	-1.703925000
1	2.828694000	0.490454000	2.290335000
1	0.647655000	-0.366107000	3.071085000
1	-1.317993000	-1.360816000	2.466661000
1	-3.535337000	3.468250000	0.281117000

Z-enol-imine IX

6	-2.718274000	0.034230000	1.890548000
6	-3.575521000	-0.818757000	1.204014000
6	-3.715162000	-0.671050000	-0.177619000
6	-3.010487000	0.319934000	-0.858033000
6	-2.121492000	1.149888000	-0.162842000
6	-1.985202000	1.013817000	1.224320000
7	-1.486017000	2.183513000	-0.879963000
6	-0.224566000	2.366290000	-0.901336000
6	0.891016000	1.545552000	-0.374447000
8	-4.576468000	-1.521593000	-0.815932000
6	2.063773000	2.181370000	0.075475000
6	3.174939000	1.428591000	0.459052000
6	3.136030000	0.043505000	0.400974000
6	1.974359000	-0.591693000	-0.040964000
6	0.863540000	0.140934000	-0.420492000
8	2.187351000	3.537991000	0.175473000
35	1.924145000	-2.506114000	-0.134447000
1	-2.616884000	-0.067344000	2.965272000
1	-4.144255000	-1.585361000	1.714853000
1	-3.140145000	0.462896000	-1.926163000
1	-1.324463000	1.675891000	1.769633000
1	0.092965000	3.262317000	-1.446571000
1	-4.590458000	-1.325646000	-1.758631000
1	4.060004000	1.946550000	0.807475000
1	3.999004000	-0.538137000	0.698294000
1	-0.025735000	-0.362763000	-0.772365000
1	1.342235000	3.974143000	0.025371000

Z-enol-imine X

6	-2.751945000	0.061106000	1.884136000
6	-3.591847000	-0.750930000	1.129202000
6	-3.676005000	-0.539393000	-0.248437000
6	-2.933804000	0.470387000	-0.857684000
6	-2.063512000	1.259904000	-0.093800000
6	-1.984892000	1.061325000	1.290999000
7	-1.389383000	2.319599000	-0.727317000
6	-0.122610000	2.457229000	-0.759856000
6	0.961074000	1.539680000	-0.329261000
8	-4.521390000	-1.349762000	-0.958524000
6	2.204580000	2.091176000	0.034516000
6	3.278335000	1.265206000	0.367217000
6	3.140122000	-0.117537000	0.330368000
6	1.916015000	-0.667568000	-0.040747000
6	0.837827000	0.142900000	-0.363356000
8	2.306751000	3.453527000	0.061622000
35	1.724274000	-2.573705000	-0.116373000
1	-2.692473000	-0.089071000	2.956469000
1	-4.188047000	-1.532072000	1.583502000
1	-3.019931000	0.659103000	-1.923278000
1	-1.340140000	1.693971000	1.888150000
1	0.237672000	3.389120000	-1.195283000
1	-4.496100000	-1.108102000	-1.890266000
1	4.228767000	1.703378000	0.656673000
1	3.976237000	-0.755660000	0.585214000
1	-0.102082000	-0.301884000	-0.656508000
1	3.209290000	3.712676000	0.275047000

Z-enol-imine XI

6	-2.773331000	0.116211000	1.875843000
6	-3.592300000	-0.710443000	1.108682000
6	-3.646347000	-0.517920000	-0.273841000
6	-2.895848000	0.486470000	-0.880478000
6	-2.050830000	1.287832000	-0.106237000
6	-2.000950000	1.109981000	1.285746000
7	-1.363433000	2.344560000	-0.729872000
6	-0.095762000	2.467500000	-0.763103000
6	0.976316000	1.535655000	-0.333583000
8	-4.434317000	-1.283870000	-1.091523000
6	2.225574000	2.071797000	0.033858000
6	3.287433000	1.232813000	0.371750000
6	3.131411000	-0.148161000	0.337466000
6	1.901550000	-0.682823000	-0.036383000
6	0.834916000	0.140566000	-0.365191000
8	2.345476000	3.432789000	0.059561000
35	1.685839000	-2.586958000	-0.106861000
1	-2.737706000	-0.021176000	2.950880000
1	-4.188665000	-1.486254000	1.578548000
1	-2.969263000	0.641205000	-1.949610000
1	-1.372203000	1.754523000	1.887188000
1	0.275687000	3.397351000	-1.193341000
1	-4.911948000	-1.932732000	-0.564505000
1	4.242502000	1.659288000	0.663434000
1	3.958362000	-0.796479000	0.596414000
1	-0.108858000	-0.292453000	-0.663482000
1	3.251486000	3.680130000	0.272218000

Z-enol-imine XII

6	-2.733591000	0.093249000	1.883093000
6	-3.566735000	-0.778763000	1.184990000
6	-3.676895000	-0.651312000	-0.201700000
6	-2.967913000	0.337265000	-0.880449000
6	-2.107272000	1.182929000	-0.175312000
6	-1.999109000	1.069216000	1.219345000
7	-1.460103000	2.215358000	-0.883836000
6	-0.196983000	2.381314000	-0.905850000
6	0.906425000	1.543542000	-0.379780000
8	-4.481503000	-1.464597000	-0.952947000
6	2.086420000	2.162111000	0.075586000
6	3.184073000	1.393086000	0.465525000
6	3.124200000	0.008581000	0.409442000
6	1.955206000	-0.609206000	-0.037263000
6	0.857635000	0.139509000	-0.424271000
8	2.230002000	3.516780000	0.175725000
35	1.875769000	-2.523189000	-0.126274000
1	-2.655572000	0.005386000	2.960910000
1	-4.131775000	-1.541304000	1.711867000
1	-3.085030000	0.444490000	-1.951421000
1	-1.356863000	1.746206000	1.768424000
1	0.132409000	3.276076000	-1.446176000
1	-4.924177000	-2.100537000	-0.381428000
1	4.075289000	1.898024000	0.817360000
1	3.976739000	-0.585737000	0.711785000
1	-0.036612000	-0.350852000	-0.782265000
1	1.393617000	3.965771000	0.015025000

Z-enol-imine XIII

6	−2.988621000	−0.604414000	1.174462000
6	−3.580542000	−1.515738000	0.298324000
6	−3.532865000	−1.265826000	−1.069340000
6	−2.913973000	−0.124652000	−1.574083000
6	−2.291646000	0.767413000	−0.693325000
6	−2.342355000	0.530455000	0.687119000
7	−1.727320000	1.949407000	−1.214232000
6	−0.521444000	2.313280000	−1.020099000
6	0.617533000	1.615427000	−0.377629000
6	1.592898000	2.367641000	0.304375000
6	2.734314000	1.748621000	0.816708000
6	2.920815000	0.383463000	0.658584000
6	1.955841000	−0.367228000	−0.014961000
6	0.816955000	0.231581000	−0.524703000
8	−3.071513000	−0.876185000	2.513443000
8	1.486595000	3.712730000	0.517468000
35	2.220986000	−2.252064000	−0.244951000
1	−4.074736000	−2.391344000	0.699811000
1	−3.999966000	−1.965318000	−1.753436000
1	−2.902439000	0.084708000	−2.636343000
1	−1.885662000	1.237278000	1.372485000
1	−0.271661000	3.293278000	−1.442566000
1	3.463486000	2.353000000	1.342138000
1	3.806984000	−0.094344000	1.055798000
1	0.082648000	−0.357968000	−1.055741000
1	−2.610249000	−0.195865000	3.014921000
1	0.626202000	4.040462000	0.235952000

Z-enol-imine XIV

6	−3.014862000	−0.570427000	1.161617000
6	−3.574327000	−1.497696000	0.280568000
6	−3.491654000	−1.266015000	−1.091874000
6	−2.868528000	−0.127978000	−1.590174000
6	−2.277846000	0.780420000	−0.699328000
6	−2.362990000	0.562681000	0.678939000
7	−1.708751000	1.960843000	−1.218030000
6	−0.502614000	2.319601000	−1.017204000
6	0.629409000	1.615071000	−0.370363000
6	1.608150000	2.362209000	0.312316000
6	2.747046000	1.737937000	0.823502000
6	2.926968000	0.371894000	0.665618000
6	1.957074000	−0.373785000	−0.005826000
6	0.821020000	0.230088000	−0.515366000
8	−3.070216000	−0.719395000	2.521334000
8	1.509051000	3.708062000	0.523428000
35	2.211626000	−2.261143000	−0.233971000
1	−4.078529000	−2.380364000	0.661274000
1	−3.934201000	−1.978591000	−1.778720000
1	−2.827954000	0.068959000	−2.654086000
1	−1.937269000	1.267109000	1.382228000
1	−0.245821000	3.299494000	−1.435089000
1	3.478345000	2.338622000	1.350135000
1	3.810512000	−0.110311000	1.063297000
1	0.083783000	−0.356409000	−1.045443000
1	−3.522662000	−1.540736000	2.739889000
1	0.636402000	4.032976000	0.279068000

Z-enol-imine XV

6	−3.064286000	−0.496350000	1.125182000
6	−3.618326000	−1.364045000	0.183076000
6	−3.482221000	−1.064547000	−1.169070000
6	−2.811729000	0.080200000	−1.593304000
6	−2.228372000	0.928978000	−0.644220000
6	−2.369329000	0.642447000	0.720688000
7	−1.615970000	2.121737000	−1.070251000
6	−0.392491000	2.420437000	−0.876493000
6	0.723404000	1.612289000	−0.324763000
6	1.802058000	2.283192000	0.282986000
6	2.909912000	1.573766000	0.746560000
6	2.971005000	0.192512000	0.602163000
6	1.912546000	−0.474216000	−0.009270000
6	0.800375000	0.218480000	−0.464899000
8	−3.231346000	−0.813452000	2.447318000
35	2.004087000	−2.376123000	−0.231816000
8	1.709284000	3.640684000	0.411532000
1	−4.152263000	−2.243140000	0.520881000
1	−3.919446000	−1.728811000	−1.906221000
1	−2.730896000	0.325417000	−2.644952000
1	−1.943253000	1.317442000	1.456377000
1	−0.099668000	3.425273000	−1.180290000
1	3.730280000	2.101791000	1.223292000
1	3.833496000	−0.354679000	0.959705000
1	−0.007957000	−0.313448000	−0.945900000
1	−2.805246000	−0.147998000	2.997425000
1	2.524933000	3.993573000	0.782395000

Z-enol-imine XVI

6	−3.052666000	−0.483262000	1.127847000
6	−3.615262000	−1.347215000	0.186548000
6	−3.487470000	−1.047522000	−1.169437000
6	−2.815625000	0.093483000	−1.591690000
6	−2.223225000	0.938484000	−0.640205000
6	−2.355818000	0.653214000	0.722638000
7	−1.608933000	2.127824000	−1.071959000
6	−0.384990000	2.424496000	−0.878966000
6	0.727500000	1.613828000	−0.324426000
6	1.801322000	2.280683000	0.295689000
6	2.903890000	1.567639000	0.766297000
6	2.964988000	0.187097000	0.615607000
6	1.911851000	−0.474780000	−0.009650000
6	0.804630000	0.221114000	−0.471720000
8	−3.149042000	−0.702500000	2.476867000
35	2.003790000	−2.376218000	−0.242495000
8	1.709136000	3.637488000	0.428187000
1	−4.155593000	−2.232180000	0.507853000
1	−3.931972000	−1.709586000	−1.904112000
1	−2.738698000	0.341601000	−2.642927000
1	−1.930122000	1.310288000	1.470512000
1	−0.089713000	3.428125000	−1.184497000
1	3.719920000	2.092302000	1.254016000
1	3.823076000	−0.362919000	0.979275000
1	−0.000181000	−0.307602000	−0.961934000
1	−3.637205000	−1.516687000	2.637318000
1	2.514861000	3.986058000	0.823889000

keto-amine form I of the *E*-enol-imine isomer

6	5.339793000	0.997910000	-0.000554000
6	5.756894000	-0.327304000	-0.000244000
6	4.795122000	-1.340572000	0.000180000
6	3.435501000	-1.033461000	0.000292000
6	3.030325000	0.307272000	-0.000067000
6	3.987289000	1.327133000	-0.000477000
7	1.677738000	0.691337000	-0.000010000
6	0.597383000	-0.082844000	-0.000121000
6	-0.700660000	0.433508000	0.000009000
6	-1.812129000	-0.463860000	-0.000115000
6	-3.083547000	0.024606000	0.000000000
6	-3.326452000	1.429051000	0.000241000
6	-2.291152000	2.319714000	0.000358000
6	-0.914135000	1.885099000	0.000253000
8	0.046569000	2.698966000	0.000353000
35	-4.585356000	-1.169069000	-0.000149000
8	5.250111000	-2.628285000	0.000523000
1	6.079321000	1.790000000	-0.000879000
1	6.805680000	-0.594966000	-0.000311000
1	2.711278000	-1.840097000	0.000705000
1	3.668448000	2.362592000	-0.000752000
1	1.420421000	1.701870000	0.000127000
1	0.739727000	-1.158696000	-0.000330000
1	-1.629709000	-1.532751000	-0.000299000
1	-4.350207000	1.784393000	0.000324000
1	-2.471315000	3.388009000	0.000536000
1	4.510807000	-3.245242000	0.000604000

keto-amine form II of the *E*-enol-imine isomer

6	5.335344000	0.999507000	-0.015816000
6	5.748465000	-0.330698000	-0.007200000
6	4.784233000	-1.341129000	0.004960000
6	3.425999000	-1.031096000	0.008331000
6	3.026604000	0.307049000	-0.001419000
6	3.986130000	1.329006000	-0.013159000
7	1.674862000	0.694443000	0.000468000
6	0.595413000	-0.081531000	-0.002467000
6	-0.702718000	0.434071000	0.000706000
6	-1.812979000	-0.465076000	-0.002771000
6	-3.084994000	0.021716000	0.000023000
6	-3.329955000	1.425760000	0.006233000
6	-2.296049000	2.318021000	0.009471000
6	-0.918298000	1.885462000	0.007010000
8	0.040739000	2.701344000	0.009582000
35	-4.585543000	-1.173739000	-0.004218000
8	5.108402000	-2.668369000	0.014656000
1	6.077652000	1.788896000	-0.025010000
1	6.804423000	-0.580847000	-0.009419000
1	2.719360000	-1.849882000	0.019614000
1	3.668619000	2.364824000	-0.020560000
1	1.417787000	1.704406000	0.004287000
1	0.740983000	-1.156794000	-0.008075000
1	-1.628604000	-1.533558000	-0.007512000
1	-4.354267000	1.779560000	0.008243000
1	-2.477933000	3.386031000	0.014074000
1	6.065657000	-2.773037000	0.011418000

keto-amine form III of the *E*-enol-imine isomer

6	-4.888758000	-1.205155000	-0.127627000
6	-5.619050000	-0.049695000	0.156837000
6	-4.935527000	1.135770000	0.396405000
6	-3.543670000	1.198981000	0.361679000
6	-2.822507000	0.040394000	0.063878000
6	-3.496969000	-1.163045000	-0.179118000
7	-1.412281000	0.019978000	0.004251000
6	-0.585481000	1.079856000	-0.046515000
6	0.793531000	1.055939000	-0.057179000
6	1.463118000	2.380726000	-0.154282000
6	2.925942000	2.339586000	-0.167774000
6	3.618218000	1.174988000	-0.099060000
6	2.930489000	-0.082758000	-0.003592000
6	1.575830000	-0.145986000	0.018457000
8	-5.593692000	-2.351791000	-0.355575000
8	0.838641000	3.443610000	-0.220303000
35	3.983276000	-1.681849000	0.100747000
1	-6.699670000	-0.100617000	0.189780000
1	-5.496037000	2.034176000	0.627153000
1	-3.042242000	2.131421000	0.580958000
1	-2.934757000	-2.061597000	-0.418989000
1	-0.996180000	-0.900781000	-0.018371000
1	-1.047911000	2.058624000	-0.096522000
1	3.436168000	3.292904000	-0.238432000
1	4.701835000	1.176429000	-0.112905000
1	1.094226000	-1.114199000	0.102656000
1	-4.994729000	-3.083633000	-0.536837000

E keto-amine form IV of the *E*-enol-imine isomer

6	-4.874451000	-1.222675000	-0.082734000
6	-5.616443000	-0.054371000	0.100682000
6	-4.943504000	1.155016000	0.255309000
6	-3.554902000	1.223844000	0.232311000
6	-2.822436000	0.046194000	0.037937000
6	-3.483204000	-1.174821000	-0.117387000
7	-1.412134000	0.027675000	0.000289000
6	-0.581476000	1.084632000	-0.031602000
6	0.797600000	1.057364000	-0.038205000
6	1.469312000	2.383232000	-0.099111000
6	2.931895000	2.340563000	-0.105563000
6	3.622180000	1.173534000	-0.061295000
6	2.932530000	-0.085304000	-0.001988000
6	1.577645000	-0.147386000	0.010186000
8	-5.451260000	-2.450208000	-0.239659000
8	0.846329000	3.448510000	-0.141907000
35	3.983180000	-1.687656000	0.064901000
1	-6.700576000	-0.089826000	0.125880000
1	-5.513683000	2.064322000	0.405350000
1	-3.064135000	2.176301000	0.375271000
1	-2.932885000	-2.096126000	-0.272582000
1	-0.999669000	-0.895048000	-0.016812000
1	-1.037950000	2.066664000	-0.063705000
1	3.443793000	3.294592000	-0.149574000
1	4.705868000	1.173792000	-0.068648000
1	1.094194000	-1.116864000	0.062272000
1	-6.410370000	-2.368918000	-0.209704000

keto-amine form V of the *E*-enol-imine isomer

6	5.325486000	0.147261000	-0.058418000
6	5.598714000	-1.220767000	0.010499000
6	4.539421000	-2.116954000	0.088367000
6	3.215654000	-1.682521000	0.097966000
6	2.951465000	-0.311615000	0.023422000
6	4.009486000	0.603038000	-0.053426000
7	1.651363000	0.225450000	0.024460000
6	0.491818000	-0.422841000	0.001208000
6	-0.737663000	0.242051000	0.012993000
6	-1.947039000	-0.517608000	-0.014355000
6	-3.152211000	0.117770000	-0.003457000
6	-3.227173000	1.539909000	0.034917000
6	-2.093778000	2.301856000	0.060636000
6	-0.778874000	1.707639000	0.050995000
8	0.271585000	2.404192000	0.072276000
35	-4.785367000	-0.888111000	-0.038480000
8	6.391433000	0.995981000	-0.131839000
1	6.627782000	-1.556389000	0.004889000
1	4.746768000	-3.179175000	0.146912000
1	2.416333000	-2.407591000	0.171877000
1	3.794967000	1.665774000	-0.111509000
1	1.504093000	1.258932000	0.045199000
1	0.513694000	-1.507301000	-0.032006000
1	-1.891743000	-1.600177000	-0.042886000
1	-4.201570000	2.013849000	0.043157000
1	-2.146719000	3.383567000	0.089278000
1	6.088743000	1.909564000	-0.170913000

keto-amine form VI of the *E*-enol-imine isomer

6	5.321986000	0.140850000	-0.124569000
6	5.584524000	-1.222235000	0.030041000
6	4.518828000	-2.103228000	0.198304000
6	3.203845000	-1.652834000	0.212437000
6	2.951744000	-0.284390000	0.045006000
6	4.011098000	0.611172000	-0.119331000
7	1.653088000	0.257407000	0.045205000
6	0.495582000	-0.394591000	-0.000539000
6	-0.738791000	0.258826000	0.025023000
6	-1.941494000	-0.510403000	-0.032442000
6	-3.151980000	0.113631000	-0.007386000
6	-3.240378000	1.533700000	0.075267000
6	-2.114561000	2.304568000	0.129182000
6	-0.793130000	1.722684000	0.106500000
8	0.249821000	2.426502000	0.150822000
35	-4.776182000	-0.905131000	-0.080843000
8	6.308641000	1.069147000	-0.289925000
1	6.605935000	-1.588621000	0.025031000
1	4.719473000	-3.160186000	0.329502000
1	2.399402000	-2.358507000	0.370265000
1	3.827227000	1.670907000	-0.248865000
1	1.508586000	1.289235000	0.086368000
1	0.525514000	-1.477284000	-0.068689000
1	-1.876424000	-1.591081000	-0.094098000
1	-4.219312000	1.997953000	0.094069000
1	-2.177256000	3.384390000	0.190693000
1	7.170275000	0.639211000	-0.283359000

keto-amine form VII of the *E*-enol-imine isomer

6	-4.627298000	-2.155123000	-0.196760000
6	-5.520602000	-1.104076000	-0.034882000
6	-5.019541000	0.189937000	0.128086000
6	-3.645808000	0.430581000	0.132109000
6	-2.760042000	-0.637908000	-0.043324000
6	-3.252238000	-1.936660000	-0.204742000
7	-1.360804000	-0.456177000	-0.054337000
6	-0.686758000	0.706439000	-0.071101000
6	0.681374000	0.881443000	-0.055841000
6	1.147525000	2.293289000	-0.100484000
6	2.599241000	2.470257000	-0.087828000
6	3.455357000	1.418916000	-0.038222000
6	2.961720000	0.070716000	0.007114000
6	1.631165000	-0.193887000	-0.000366000
8	0.371640000	3.253556000	-0.145059000
35	4.241353000	-1.354819000	0.083551000
8	-5.929494000	1.192371000	0.291855000
1	-5.005503000	-3.162768000	-0.323160000
1	-6.591363000	-1.261313000	-0.029664000
1	-3.283572000	1.440554000	0.281834000
1	-2.566434000	-2.765436000	-0.342668000
1	-0.824521000	-1.313037000	-0.065475000
1	-1.275997000	1.614796000	-0.109182000
1	2.963486000	3.490224000	-0.121022000
1	4.526691000	1.581413000	-0.030725000
1	1.296170000	-1.224706000	0.040402000
1	-5.482417000	2.041229000	0.378822000

keto-amine form VIII of the *E*-enol-imine isomer

6	-4.608463000	-2.151836000	-0.214610000
6	-5.508237000	-1.103783000	-0.040195000
6	-5.014254000	0.190895000	0.137072000
6	-3.642548000	0.440488000	0.141617000
6	-2.754564000	-0.619231000	-0.045740000
6	-3.238484000	-1.923165000	-0.221247000
7	-1.356547000	-0.433277000	-0.058471000
6	-0.683349000	0.730989000	-0.071188000
6	0.685837000	0.898122000	-0.057105000
6	1.165233000	2.305686000	-0.105419000
6	2.619297000	2.469559000	-0.090488000
6	3.466228000	1.411314000	-0.038762000
6	2.959863000	0.067692000	0.007939000
6	1.627198000	-0.185021000	0.000145000
8	0.399677000	3.272742000	-0.155585000
35	4.226570000	-1.369629000	0.088508000
8	-5.828918000	1.269374000	0.322366000
1	-4.982034000	-3.159788000	-0.351548000
1	-6.577385000	-1.288340000	-0.038373000
1	-3.309045000	1.456625000	0.300988000
1	-2.547115000	-2.745646000	-0.368528000
1	-0.817734000	-1.288212000	-0.078709000
1	-1.273660000	1.639046000	-0.104925000
1	2.991978000	3.486454000	-0.124756000
1	4.538997000	1.564100000	-0.030351000
1	1.284229000	-1.213111000	0.044176000
1	-6.750972000	0.991638000	0.317184000

keto-amine form I of the *Z*-enol-imine isomer

6	4.076161000	-0.387642000	-1.166882000
6	3.972915000	-1.173458000	-0.026765000
6	3.048186000	-0.826267000	0.962855000
6	2.241358000	0.300296000	0.811349000
6	2.341670000	1.066403000	-0.353040000
6	3.262686000	0.731546000	-1.345105000
7	1.564330000	2.251103000	-0.504902000
6	0.261028000	2.488097000	-0.242829000
6	-0.814650000	1.648273000	-0.029439000
6	-0.824345000	0.221183000	-0.214062000
6	-1.981820000	-0.474260000	-0.084203000
6	-3.220739000	0.173670000	0.241526000
6	-3.269473000	1.516965000	0.421644000
6	-2.079162000	2.359878000	0.321084000
8	-2.129537000	3.577645000	0.523020000
35	-2.010332000	-2.373658000	-0.362247000
8	2.993301000	-1.619464000	2.070695000
1	4.790996000	-0.655291000	-1.936135000
1	4.591082000	-2.050790000	0.115456000
1	1.543515000	0.587921000	1.589841000
1	3.330692000	1.328504000	-2.246812000
1	2.094506000	3.061748000	-0.791198000
1	0.011165000	3.545922000	-0.214303000
1	0.083497000	-0.295880000	-0.488533000
1	-4.116896000	-0.428489000	0.335889000
1	-4.198233000	2.019565000	0.664373000
1	2.288002000	-1.326443000	2.657862000

keto-amine form II of the *Z*-enol-imine isomer

6	4.029002000	-0.354896000	-1.219051000
6	3.966598000	-1.136980000	-0.069302000
6	3.079159000	-0.787158000	0.952415000
6	2.261846000	0.335027000	0.824393000
6	2.322907000	1.095797000	-0.341636000
6	3.210505000	0.760863000	-1.368163000
7	1.535249000	2.276615000	-0.473521000
6	0.227850000	2.495327000	-0.213677000
6	-0.839018000	1.642838000	-0.011504000
6	-0.828314000	0.214620000	-0.187596000
6	-1.979682000	-0.493305000	-0.076927000
6	-3.231319000	0.140747000	0.225994000
6	-3.298884000	1.483732000	0.402091000
6	-2.117574000	2.340624000	0.316768000
8	-2.186696000	3.558943000	0.510032000
35	-1.981469000	-2.394384000	-0.346567000
8	2.974451000	-1.494514000	2.113824000
1	4.716071000	-0.625903000	-2.012005000
1	4.601924000	-2.010657000	0.035317000
1	1.595418000	0.602343000	1.634112000
1	3.244486000	1.356129000	-2.272773000
1	2.056118000	3.095865000	-0.752051000
1	-0.035000000	3.549904000	-0.181575000
1	0.091465000	-0.293917000	-0.435977000
1	-4.121605000	-0.471823000	0.308334000
1	-4.237088000	1.975695000	0.629826000
1	3.572377000	-2.249345000	2.097605000

keto-amine form III of the *Z*-enol-imine isomer

6	4.964962000	-0.029241000	0.063259000
6	4.770220000	0.960850000	-0.901547000
6	3.522045000	1.090208000	-1.498134000
6	2.464079000	0.252561000	-1.154091000
6	2.670571000	-0.729698000	-0.189210000
6	3.920025000	-0.878650000	0.418590000
7	1.665929000	-1.682928000	0.134028000
6	0.319178000	-1.603703000	0.137026000
6	-0.554376000	-0.533681000	0.262535000
6	-0.160839000	0.809644000	0.745699000
6	-1.228207000	1.805010000	0.772214000
6	-2.517006000	1.500972000	0.469926000
6	-2.879321000	0.165836000	0.097668000
6	-1.945880000	-0.813484000	0.006455000
8	6.206360000	-0.118307000	0.631029000
8	0.981553000	1.067587000	1.142648000
35	-4.725281000	-0.206689000	-0.272184000
1	5.594230000	1.612857000	-1.162381000
1	3.367597000	1.858726000	-2.246485000
1	1.499859000	0.355128000	-1.632511000
1	4.062760000	-1.634014000	1.185925000
1	2.021837000	-2.625583000	0.207186000
1	-0.141258000	-2.584137000	0.036217000
1	-0.943380000	2.805752000	1.075123000
1	-3.288732000	2.260165000	0.518960000
1	-2.232581000	-1.817630000	-0.285632000
1	6.206981000	-0.790650000	1.320078000

keto-amine form IV of the *Z*-enol-imine isomer

6	4.988295000	-0.075121000	0.048128000
6	4.787149000	0.956359000	-0.870905000
6	3.524924000	1.133280000	-1.432140000
6	2.463275000	0.303782000	-1.092287000
6	2.677603000	-0.718396000	-0.166414000
6	3.936270000	-0.915530000	0.400547000
7	1.665922000	-1.662268000	0.153850000
6	0.319663000	-1.584722000	0.155740000
6	-0.560471000	-0.517794000	0.269248000
6	-0.179592000	0.830910000	0.746646000
6	-1.255329000	1.817428000	0.765376000
6	-2.540646000	1.502296000	0.459374000
6	-2.890622000	0.162870000	0.091861000
6	-1.948840000	-0.809409000	0.009691000
8	6.197793000	-0.312781000	0.641949000
8	0.959742000	1.101500000	1.144595000
35	-4.731918000	-0.226418000	-0.284293000
1	5.607142000	1.612979000	-1.144199000
1	3.368085000	1.932309000	-2.147112000
1	1.490988000	0.440266000	-1.543905000
1	4.106019000	-1.696213000	1.132834000
1	2.022376000	-2.602556000	0.251814000
1	-0.137111000	-2.567917000	0.066221000
1	-0.979628000	2.821533000	1.065544000
1	-3.318482000	2.255586000	0.502404000
1	-2.226730000	-1.817313000	-0.277839000
1	6.832660000	0.356371000	0.365999000

Z keto-amine form V of the *Z*-enol-imine isomer

6	3.958169000	-0.480374000	-0.405447000
6	3.780949000	-1.106845000	0.829404000
6	2.813581000	-0.627521000	1.705416000
6	2.021149000	0.470344000	1.375541000
6	2.197320000	1.080241000	0.134067000
6	3.164165000	0.612028000	-0.758731000
7	1.445985000	2.241309000	-0.222155000
6	0.113611000	2.457663000	-0.185031000
6	-0.965152000	1.604667000	-0.065926000
6	-0.914856000	0.166575000	-0.085636000
6	-2.063056000	-0.554938000	-0.071759000
6	-3.351775000	0.075874000	-0.028989000
6	-3.457246000	1.427822000	-0.009053000
6	-2.284683000	2.300770000	-0.008833000
8	-2.393484000	3.530608000	0.040117000
35	-2.005603000	-2.473214000	-0.131211000
8	4.918556000	-0.985934000	-1.232968000
1	4.402421000	-1.956555000	1.082415000
1	2.681383000	-1.109584000	2.666915000
1	1.280359000	0.850796000	2.066759000
1	3.280218000	1.088788000	-1.727263000
1	2.003092000	3.048045000	-0.464920000
1	-0.158036000	3.505195000	-0.290574000
1	0.036651000	-0.341804000	-0.136479000
1	-4.238877000	-0.546712000	-0.016142000
1	-4.423589000	1.917042000	0.021926000
1	4.936963000	-0.497092000	-2.062510000

keto-amine form VI of the *Z*-enol-imine isomer

6	4.025025000	-0.465634000	-0.375430000
6	3.796343000	-1.125060000	0.833190000
6	2.775096000	-0.682602000	1.672049000
6	1.982706000	0.405846000	1.325822000
6	2.210593000	1.046274000	0.103925000
6	3.229073000	0.619025000	-0.745743000
7	1.461416000	2.201458000	-0.265355000
6	0.132956000	2.438006000	-0.210380000
6	-0.957140000	1.602406000	-0.068638000
6	-0.931355000	0.164196000	-0.103336000
6	-2.091121000	-0.538205000	-0.073786000
6	-3.368230000	0.112981000	-0.000474000
6	-3.450595000	1.466253000	0.032673000
6	-2.263422000	2.319312000	0.020194000
8	-2.350146000	3.550061000	0.085589000
35	-2.067185000	-2.456600000	-0.155987000
8	5.006305000	-0.836833000	-1.248695000
1	4.411739000	-1.972552000	1.117974000
1	2.605394000	-1.187877000	2.615541000
1	1.204118000	0.759007000	1.988980000
1	3.403873000	1.104277000	-1.698482000
1	2.022666000	2.989467000	-0.555731000
1	-0.124020000	3.488695000	-0.320962000
1	0.009895000	-0.359412000	-0.183344000
1	-4.265131000	-0.495050000	0.023557000
1	-4.407782000	1.971326000	0.085852000
1	5.471966000	-1.609686000	-0.911896000

keto-amine form VII of the *Z*-enol-imine isomer

6	5.152902000	-0.650540000	0.290538000
6	5.057111000	0.630285000	-0.242898000
6	3.823611000	1.080852000	-0.713798000
6	2.698983000	0.259688000	-0.662669000
6	2.808180000	-1.013129000	-0.106112000
6	4.037836000	-1.478862000	0.366560000
7	1.716212000	-1.918675000	-0.099539000
6	0.379172000	-1.751051000	-0.131948000
6	-0.456118000	-0.698005000	0.219975000
6	-0.049302000	0.453101000	1.056102000
6	-1.081927000	1.457852000	1.287716000
6	-2.359188000	1.307879000	0.849498000
6	-2.741713000	0.136067000	0.121085000
6	-1.837493000	-0.831278000	-0.172708000
8	1.076243000	0.547308000	1.562088000
35	-4.571296000	-0.037121000	-0.430509000
8	3.772391000	2.344595000	-1.232419000
1	6.107081000	-1.004194000	0.663172000
1	5.914434000	1.289415000	-0.293564000
1	1.752349000	0.604967000	-1.058305000
1	4.113284000	-2.467356000	0.805579000
1	2.007790000	-2.875187000	-0.244742000
1	-0.122034000	-2.644543000	-0.497584000
1	-0.783948000	2.327823000	1.861248000
1	-3.106321000	2.064622000	1.058225000
1	-2.139880000	-1.710218000	-0.730987000
1	2.857726000	2.592252000	-1.403849000

keto-amine form VIII of the *Z*-enol-imine isomer

6	5.110060000	-0.607289000	0.353986000
6	5.011279000	0.669367000	-0.196015000
6	3.790982000	1.093507000	-0.723389000
6	2.678135000	0.255761000	-0.702976000
6	2.791484000	-1.010780000	-0.140204000
6	4.010131000	-1.454309000	0.385473000
7	1.712475000	-1.932618000	-0.170857000
6	0.374236000	-1.766027000	-0.207148000
6	-0.454491000	-0.727066000	0.191158000
6	-0.035937000	0.381357000	1.079494000
6	-1.056449000	1.388984000	1.350755000
6	-2.334047000	1.271814000	0.905418000
6	-2.729644000	0.134528000	0.129168000
6	-1.837066000	-0.829051000	-0.207097000
8	1.086363000	0.434508000	1.596311000
35	-4.561998000	0.005284000	-0.426701000
8	3.620282000	2.326803000	-1.288498000
1	6.054752000	-0.938870000	0.768644000
1	5.872848000	1.329367000	-0.211608000
1	1.749130000	0.603437000	-1.132282000
1	4.083867000	-2.438615000	0.833529000
1	2.012339000	-2.880311000	-0.351432000
1	-0.126197000	-2.640374000	-0.617567000
1	-0.747315000	2.233487000	1.955322000
1	-3.072336000	2.029048000	1.141917000
1	-2.148313000	-1.679180000	-0.803787000
1	4.438636000	2.830138000	-1.226442000

Table S2. B3LYP/6-311++G(d,p) calculated infrared spectra for conformers I, II, V and VI of BHAP (*E*-enol-imine isomer).[a]

ν	I^{IR}	ν	I^{IR}	ν	I^{IR}	ν	I^{IR}
I		II		V		VI	
22.7	0.17	23.1	0.36	25.7	0.10	26.1	0.12
45.9	0.50	46.1	0.18	49.1	0.17	49.1	0.48
51.0	0.10	50.9	0.63	56.0	0.14	55.5	0.88
110.6	0.79	110.6	1.88	109.0	1.02	108.8	1.65
140.0	0.58	140.1	0.71	137.7	0.42	137.5	0.69
156.5	0.38	156.6	1.50	151.6	0.66	151.7	0.53
183.5	1.11	183.8	1.86	196.0	1.60	195.2	1.86
227.9	1.96	228.8	1.08	231.3	3.12	232.2	1.34
266.0	4.51	266.3	3.89	262.4	4.60	261.8	2.97
274.4	2.5	274.4	5.81	274.0	2.81	273.5	3.10
292.6	0.39	292.5	1.82	293.5	0.05	293.1	1.37
299.3	104.68	318.1	102.16	314.7	6.79	315.9	4.05
327.7	2.68	327.8	1.83	320.0	103.91	317.9	103.87
348.6	0.36	347.9	1.45	347.4	0.64	347.6	0.56
401.2	11.65	402.0	2.11	404.9	8.06	403.9	5.97
448.8	0.87	449.6	5.45	449.5	1.10	449.8	4.38
461.3	6.33	462.5	7.23	457.7	1.12	458.0	5.27
474.4	13.39	476.1	7.31	477.3	15.91	478.2	12.07
520.6	10.73	520.8	2.58	516.2	17.41	514.8	1.09
533.7	0.15	533.7	1.35	530.8	1.48	531.3	0.95
555.9	5.30	555.2	6.08	552.7	4.79	553.3	4.07
562.6	12.23	563.8	9.94	574.1	10.14	573.9	16.78
620.7	5.17	623.8	5.69	624.7	8.06	624.2	5.90
634.6	20.74	635.4	22.21	635.8	16.53	635.4	22.32
684.1	15.67	685.2	15.16	686.0	16.75	685.1	16.35
692.4	21.88	694.3	22.88	695.7	25.80	694.9	22.62
729.0	1.24	728.8	1.49	729.0	1.13	728.3	1.94
779.5	9.55	775.9	34.01	774.9	6.42	766.5	31.46
786.7	22.94	779.0	11.85	780.4	20.54	777.2	6.29
802.2	8.92	805.0	7.86	813.2	7.16	812.8	9.62
827.8	84.76	828.0	80.95	827.8	83.95	827.5	85.23
837.6	23.13	851.8	16.90	848.8	4.55	849.8	16.98
851.3	27.92	866.3	12.84	856.2	42.42	860.0	1.39
881.7	9.99	874.1	8.22	880.0	5.20	878.3	4.30
890.6	3.19	884.3	17.90	883.7	9.64	884.3	26.64
916.6	13.75	917.1	16.23	919.5	17.42	919.7	22.58
958.8	0.40	958.4	0.46	958.6	0.04	958.4	0.17
978.5	47.35	970.2	1.72	969.8	53.61	959.8	0.15
980.3	3.94	978.2	94.91	973.5	0.10	967.3	102.62
998.9	8.19	998.2	9.06	994.4	10.83	994.6	12.87
1006.0	3.20	1007.9	2.18	1005.4	7.15	1004.4	5.38
1085.2	16.57	1085.2	15.91	1084.1	15.73	1084.0	15.40
1090.0	10.15	1097.4	10.81	1095.7	13.00	1104.3	7.41
1144.7	7.80	1144.6	8.91	1144.6	7.08	1144.6	11.99
1159.9	192.64	1156.5	192.27	1158.5	160.77	1147.9	182.24
1182.3	77.39	1175.7	36.17	1178.0	28.77	1178.2	18.14
1184.2	94.52	1194.6	41.45	1186.9	169.23	1196.6	61.24
1213.1	31.42	1215.4	57.53	1215.4	56.05	1216.7	52.27
1250.4	18.13	1250.8	17.94	1249.8	9.74	1249.6	19.41
1284.5	81.72	1282.7	47.47	1292.9	28.68	1291.5	36.08
1304.6	81.83	1304.2	79.51	1302.9	131.61	1303.0	85.34
1328.1	2.49	1318.7	92.19	1326.2	1.70	1316.5	137.51
1335.1	1.71	1334.9	5.00	1334.8	2.83	1334.6	6.65
1338.3	9.73	1338.8	16.45	1338.8	2.96	1339.0	2.24
1372.7	47.63	1373.8	34.526	1371.9	40.64	1371.7	17.22
1418.6	34.09	1419.1	31.19	1418.2	25.92	1417.8	51.02
1467.7	44.47	1469.6	15.53	1464.1	52.23	1466.7	16.00
1469.7	29.51	1485.5	10.71	1478.2	87.27	1489.5	81.19
1495.1	132.13	1494.6	156.07	1496.9	81.64	1497.4	37.28
1518.0	60.17	1507.4	87.04	1514.8	63.04	1504.5	126.55
1588.4	159.41	1589.0	141.37	1588.5	173.04	1588.8	153.68
1617.5	194.62	1612.2	114.30	1617.6	80.99	1608.1	67.49
1623.5	84.36	1627.3	245.11	1623.9	272.72	1629.3	346.86
1644.3	17.74	1644.7	14.50	1644.6	23.95	1644.7	28.059
1656.2	144.18	1658.1	117.52	1656.5	97.93	1660.4	75.63
2872.2	37.41	2877.4	34.47	2878.3	34.13	2876.2	34.61
2985.2	7.50	2983.9	10.30	2991.0	7.78	2983.8	9.26
3001.4	10.11	3005.8	17.87	2998.2	8.52	3002.4	14.72
3008.9	0.98	3009.9	1.38	3008.2	1.29	3008.0	1.12
3014.9	2.28	3014.8	4.04	3014.4	4.46	3014.5	3.23
3020.8	6.34	3019.4	1.97	3015.9	4.44	3018.1	8.41
3026.5	4.32	3023.9	5.01	3025.6	11.12	3025.2	1.68
3028.5	2.59	3028.4	4.16	3027.7	2.81	3027.8	2.41
3040.3	533.91	3033.6	538.05	3033.1	536.05	3036.8	546.02
3623.9	55.08	3625.1	84.34	3622.9	66.48	3625.4	87.62

[a] Frequencies (ν, in cm^{-1}) have been scaled by 0.994 or 0.945 below and above 1800 cm^{-1}, respectively. Infrared intensities (I^{IR}) in km mol^{-1}.
